# Drought Tolerant *Enterobacter* sp./*Leclercia adecarboxylata* Secretes Indole-3-acetic Acid and Other Biomolecules and Enhances the Biological Attributes of *Vigna radiata* (L.) R. Wilczek in Water Deficit Conditions

**DOI:** 10.3390/biology10111149

**Published:** 2021-11-08

**Authors:** Bilal Ahmed, Mohammad Shahid, Asad Syed, Vishnu D. Rajput, Abdallah M. Elgorban, Tatiana Minkina, Ali H. Bahkali, Jintae Lee

**Affiliations:** 1 School of Chemical Engineering, Yeungnam University, Gyeongsan 38541, Korea; 2 Department of Agricultural Microbiology, Faculty of Agricultural Sciences, Aligarh Muslim University, Aligarh 202002, India; gd4858@myamu.ac.in; 3 Department of Botany and Microbiology, College of Science, King Saud University, P.O. Box 2455, Riyadh 11451, Saudi Arabia; assyed@ksu.edu.sa (A.S.); aelgorban@ksu.edu.sa (A.M.E.); abahkali@ksu.edu.sa (A.H.B.); 4 Academy of Biology and Biotechnology, Southern Federal University, 344090 Rostov-on-Don, Russia; rvishnu@sfedu.ru (V.D.R.); tminkina@mail.ru (T.M.)

**Keywords:** drought stress, *Enterobacter* sp./*L. adecarboxylata*, growth regulating substances, *Vigna radiata* (L.) R. Wilczek, growth improvement, gas exchange parameters

## Abstract

**Simple Summary:**

Drought stress is one of the most important factors that significantly affects agricultural lands and reduces the production of various crops. Among bean crops, *Vigna radiata* (mung bean) is a highly nutritious food crop that provides protein, carbohydrates, several essential minerals, amino acids, vitamins, and antioxidants. To resolve the problem of drought-affected agriculture of mung bean, we focused on finding a novel and sustainable solution by using a drought-tolerant bacterium *Enterobacter* sp./*Leclercia adecarboxylata* PAB19 that produces significant amounts of plant growth-promoting bioactive compounds and colonizes the roots of mung bean plants. By performing a series of in vitro and in situ (on field) experiments, we conclude that the bacterium PAB19 holds a great potential to mitigate drought stress. Several agriculturally important parameters were enhanced by the bacterial activity which was suppressed by the drought stress induced by a chemical PEG-6000 without bacterial inoculation. Conclusively, strain PAB19 can be applied to alleviate drought stress by improving the biological attributes of mung bean under water-deficit conditions.

**Abstract:**

Drought or water stress is a limiting factor that hampers the growth and yield of edible crops. Drought-tolerant plant growth-promoting rhizobacteria (PGPR) can mitigate water stress in crops by synthesizing multiple bioactive molecules. Here, strain PAB19 recovered from rhizospheric soil was biochemically and molecularly characterized, and identified as *Enterobacter* sp./*Leclercia adecarboxylata* (MT672579.1). Strain PAB19 tolerated an exceptionally high level of drought (18% PEG-6000) and produced indole-3-acetic acid (176.2 ± 5.6 µg mL^−1^), ACC deaminase (56.6 ± 5.0 µg mL^−1^), salicylic acid (42.5 ± 3.0 µg mL^−1^), 2,3-dihydroxy benzoic acid (DHBA) (44.3 ± 2.3 µg mL^−1^), exopolysaccharide (204 ± 14.7 µg mL^−1^), alginate (82.3 ± 6.5 µg mL^−1^), and solubilized tricalcium phosphate (98.3 ± 3.5 µg mL^−1^), in the presence of 15% polyethylene glycol. Furthermore, strain PAB19 alleviated water stress and significantly (*p* ≤ 0.05) improved the overall growth and biochemical attributes of *Vigna radiata* (L.) R. Wilczek. For instance, at 2% PEG stress, PAB19 inoculation maximally increased germination, root dry biomass, leaf carotenoid content, nodule biomass, leghaemoglobin (LHb) content, leaf water potential (ΨL), membrane stability index (MSI), and pod yield by 10%, 7%, 14%, 38%, 9%, 17%, 11%, and 11%, respectively, over un-inoculated plants. Additionally, PAB19 inoculation reduced two stressor metabolites, proline and malondialdehyde, and antioxidant enzymes (POD, SOD, CAT, and GR) levels in *V. radiata* foliage in water stress conditions. Following inoculation of strain PAB19 with 15% PEG in soil, stomatal conductance, intercellular CO_2_ concentration, transpiration rate, water vapor deficit, intrinsic water use efficiency, and photosynthetic rate were significantly improved by 12%, 8%, 42%, 10%, 9% and 16%, respectively. Rhizospheric CFU counts of PAB19 were 2.33 and 2.11 log CFU g^−1^ after treatment with 15% PEG solution and 8.46 and 6.67 log CFU g^−1^ for untreated controls at 40 and 80 DAS, respectively. Conclusively, this study suggests the potential of *Enterobacter* sp./*L. adecarboxylata* PAB19 to alleviate water stress by improving the biological and biochemical features and of *V. radiata* under water-deficit conditions.

## 1. Introduction

Crops face a wide range of environmental stresses [1,2], and drought stress now poses a major obstacle to global crop production sustainability [3]. Climate simulations predict drought stress will continue and become more severe [4,5], and it has been predicted that irrigation water demands will hugely increase by 2050 [6]. Drought stress affects plants morphologically, physiologically, and biochemically due to the generation of reactive oxygen species (ROS) [7,8]. Many approaches have been implemented recently to increase the drought tolerance of crops, such as conventional breeding and genetic engineering. However, these methods have certain limitations that prevent their utilization in agricultural practices [9]. In this regard, drought-tolerant rhizobacteria that promote plant development are considered a viable option for sustainable agriculture in water-scarce areas. *Vigna radiata* (mung bean), a widely grown nutrient-dense grain legume in the tropics, is noted for its detoxifying properties and is used to relieve heat exhaustion and minimize swelling in summer [10]. 

Beneficial rhizobacteria, often referred to as plant growth-promoting rhizobacteria (PGPR), have an important role in plant development, nutrition management, and biocontrol activities [11,12,13]. These PGPR invade plant rhizospheres/endo-rhizospheres and stimulate plant development by one or more direct and/or indirect methods [14,15,16,17]. In addition, the relevance of PGPR for the control of biotic and abiotic stressors is growing. The mechanism of drought tolerance and its mitigation for plants by rhizobacteria might be a collective outcome of several activities including (i) the synthesis of phytohormones such as indole-3-acetic acid (IAA), abscisic acid (ABA), gibberellic acid, and cytokinins; (ii) production of ACC deaminase decreasing the ethylene levels in roots; (iii) by releasing exopolysaccharides; and (iv) induction of systemic tolerance to diseases by bacterial compounds [18]. Recently, PGPR were found to contribute to plant abiotic stress management [2,19]. Many recent studies have demonstrated the importance of PGPR, such as *Ochrobactrum* sp. [20] and *Pseudomonas pseudoalcaligenes* [21], for the amelioration of drought stress in edible crops. For example, when drought-tolerant PGPR strains were administered as soil inoculants to maize plants grown in a water deficit environment, they significantly improved the growth, root elongation, and NPK uptake by reducing ethylene levels in plants [22]. Similarly, root and shoot lengths, stem diameters, dry biomass, and chlorophyll contents of bean plants cultivated in drought-stressed soils were augmented following the application of the drought-tolerant PGPR strain *Rhodobacter sphaeroides* KE149 [23]. 

By affecting the biochemical (antioxidative defense system) and physiological (photosynthetic attributes) systems, drought-tolerant PGPR protect crops from abiotic stress [24,25]. In the present study, the drought-tolerant PGPR strain *Enterobacter* sp./*L. adecarboxylata* PAB19 was isolated from rhizosphere soil, characterized and applied to *V. radiata*, considering its ability to endure water stress and to produce multiple bioactive metabolites including IAA, ACC deaminase, siderophores, and ammonia. Additionally, the biofilm-forming ability of the strain was assessed. The abilities of PGPR to synthesize growth-regulating substances and form biofilms are believed to protect microbial cells from stress and help them withstand constantly changing environmental conditions in the rhizosphere. Despite their global importance, firm data on the effect of water stress on legumes, especially *V. radiata* is scarce. Therefore, our goals were: (i) to isolate and identify drought-tolerant PGPR strains with multiple PGP features (this led to the selection of strain PAB19); (ii) to assess the effect of PEG-6000 (most effective water-repellent agents) on plant growth regulating substances in strain PAB19; (iii) to evaluate the mitigation potential of PAB19 on growth, dry biomass, leaf pigments, and nutrient uptake of *V. radiata* cultivated under water deficit conditions; (iv) to determine the effects of PAB19 on rhizobia-*V. radiata* symbiosis and seed attributes; (v) to assess stress markers and antioxidant response in inoculated plants raised under water deficit conditions; and (vi) to evaluate the effects of PAB19 on leaf exchange parameters of treated and untreated *V. radiata* plants.

## 2. Material and Methods

### 2.1. Isolation and Biochemical Characterization of Bacterial Isolates

To isolate PGPR, soil samples were collected from the rhizosphere region of fields cultivating vegetables from the agricultural fields of Faculty of Agricultural Sciences, Aligarh Muslim University, Aligarh, India (coordinates: 27.92249, 78.07141). The soils were serially diluted and 100 µL aliquots were spread plated on Pikovskaya (PVK) agar (g L^−1^: glucose 10; Ca_3_(PO_4_)_2_ 5; (NH_4_)_2_SO_4_ 0.5; NaCl 0.2; MgSO_4_⋅7H_2_O 0.1; KCl 0.1; yeast extract 0.5; MnSO_4_ and FeSO_4_ trace; agar 15; pH 7.0) medium. Plates were incubated for 4–5 days at 28 ± 2 °C. Colonies with various zones of solubilization (halo zones) around bacterial growth were purified three times on the same medium. These isolates were subsequently examined by Gram’s staining and biochemically and morphologically characterized following earlier described methods [26]. Starch and gelatin hydrolysis by isolates were also evaluated [26]. 

### 2.2. Drought Tolerance Assay of Bacterial Isolates 

To assess the water stress tolerance of isolated PGPR strains, we used minimum inhibitory concentrations (MICs) against polyethylene glycol (PEG-6000) (Himedia, Pvt., Ltd., Mumbai, India). Briefly, PEG amended (0–20%) PKV agar plates were prepared and spot inoculated with 10 μL of freshly grown cultures. Plates were incubated at 28 ± 2 °C for two days and growth was recorded. 

### 2.3. Molecular Characterization of Strain PAB19

After morphological and biochemical characterization and determining its tolerance to abiotic stress (PEG), a region of the gene encoding the 16S rRNA of the strain PAB19 was amplified by PCR and sequenced using a commercially available service provided by Macrogen, Seoul, South Korea (refer to Section S2.3). A phylogenetic tree was constructed in order to identify strain PAB19.

### 2.4. Plant Growth Regulating (PGR) Substance Release by Enterobacter sp./L. adecarboxylata PAB19 under Different Levels of Water Stress

#### 2.4.1. Quantification of Indole-3-acetic Acid (IAA)

The IAA synthesized by PAB19 was quantitatively evaluated as described by Bric et al. [27]. For this assessment, PAB19 was cultured in Luria Bertani (LB) broth (HiMedia Pvt. Ltd. Mumbai, India) containing a fixed concentration (100 µg/mL) of tryptophan and various concentrations of PEG-6000 (0–15%). The culture was incubated at 28 ± 2 °C at 125 rpm for 2–3 days. After completion of incubation, 5.0 mL of culture was centrifuged at 10,000× *g* for 20 min and 2.0 mL of supernatant was mixed with 4.0 mL of Salkowski reagent and few drops of orthophosphoric acid were added. After adding these reagents, tubes were kept for 30 min in darkness. The absorbance of the pink solution was read at 530 nm using UV–Vis spectrophotometer (UV-2450, Shimadzu, Tokyo, Japan). 

#### 2.4.2. Bioassay of Siderophore Production

The PGPR isolates were spot inoculated on 0–15% PEG supplemented universal chrome azurol S (CAS) agar plates and then siderophore production was investigated as previously described [28,29]. Furthermore, phenolate siderophore (SA and 2,3-DHBA) synthesized by strain PAB19 in the absence or presence of different levels of the drought was estimated following the method of Reeves et al. (1983) (refer to Section S2.4.2).

#### 2.4.3. ACC Deaminase Activity

The ACC deaminase activity of strain PAB19 was assessed by culturing bacterial cells in a liquid medium supplemented with different concentrations (0–15%) of PEG solution [30,31] (refer to Section S2.4.3).

#### 2.4.4. P-Solubilization and NH_3_ Production

The P-solubilization ability of PAB19 was examined quantitatively by growing the bacterium in Pikovskaya’s broth containing different concentrations of PEG-6000 [32]. After inoculation, flasks were incubated at 30 ± 2 °C for seven days on a rotary shaker at 120 rpm. The quantity of solubilized P as a result of bacterial activity was measured using chlorostannous-reduced molybdo-phosphoric acid after removing 10 mL of culture broth from each flask and centrifuged at 5724× *g* for 30 min. A volume of 100 µL of chlorostannous acid was added to 10 mL supernatant, and the content was adjusted to 50 mL with distilled water. The blue colour developed was read at 600 nm on UV-visible spectrophotometer. Using the potassium dihydrogen phosphate (KH_2_PO_4_) calibration curve, the amount of P-solubilized was determined. Hydrogen cyanide and ammonia synthesis by PAB19 were assayed following the methods described by Bakker and Schippers [33] and Dye [34], respectively. 

### 2.5. Assessment of Biofilm Development and Associated Traits by PAB19 under Water Stress

The development of biofilms on 96 well-plates by PAB19 in the absence/presence of PEG was assessed using 1% crystal violet (CV) as previously described [35] (refer to Section S2.5). Extracellular polymeric substances (EPS) produced by the strain in the presence of water stress were estimated as described by Mody et al. [36]. Alginate produced by strain PAB19 was also assayed. For this assay, cells were grown in a liquid medium containing different concentrations of PEG (Section S2.5). Similarly, the cell surface hydrophobicity (CSH) of PAB19 was quantified by cultivating cells with/without added PEG using the bacterial adhesion to hydrocarbons (BATH) method previously described by Rosenberg [37]. 

### 2.6. Plant Experiments

#### 2.6.1. Bacterial Inoculation, Plant Culture, and Seed Treatment

Seeds of *V. radiata* (L.) R. Wilczek were procured from a local market, surface sterilized with 3% sodium hypochlorite (NaOCl), washed thrice, and dried at room temperature. Bacterial coating of seeds was performed using freshly prepared inoculums of ~1 × 10^9^ cells. Polyethylene glycol (PEG-6000) solutions were prepared at 2, 5, 10, or 15% in double-distilled water (DDW) and applied to make the soil wet at least one day before sowing. Treated soils (5 kg per earthen pot; 20 × 24 cm in diameter) were prepared. The pots were set up in a random block configuration and the experiment was performed in triplicate. Seedlings were thinned after germination, and two healthy *V. radiata* seedlings of similar lengths were cultivated in each pot for 15 days after emergence (DAE). The pots were watered regularly and kept in an open field environment (~9 h light/~15 h dark). To ensure the consistency of our findings, crop trials were also repeated in the consecutive year under similar environmental conditions.

#### 2.6.2. Germination Efficiency, Vigor Indices, Leaf Water Potential, and Membrane Stability Indices

##### Seed Germination Efficiencies and Seedling Vigor Indices (SVIs)

Surface sterilized (3% NaOCl, 10 min) seeds of *V. radiata* were coated with *Enterobacter* sp./*L. adecarboxylata* (~1 × 10^9^ cells) and sown in earthen pots containing 5.0 kg soils pre-treated with different concentrations of stressor, i.e., PEG-6000. Six days after sowing (DAS), the seed germination efficiency was recorded. Seed germination efficiencies (%) were calculated as follows:

$$Germination\;\%=\frac{Number\;of\;germinated\;seeds}{Total\;number\;of\;seeds}\times 100$$

SVI were calculated using the following formula:

SVI = [Root length + Shoot length] × % Seed Germination

##### Leaf Water Potential (ΨL) Assessment

The ΨL of the youngest fully expanded leaves of PEG-treated and strain PAB19 inoculated *V. radiata* plants were estimated using a pressure chamber (model 615, PMS Instruments, Albany, OR, USA).

##### Membrane Stability Index (MSI)

The MSI of *V. radiata* plants grown on PEG-treated soils and inoculated with strain PAB19 was determined. For the assay, 0.1 gm leaf samples were placed in glass vials containing 10 mL of DDW and then placed in a water bath at 40 °C for 30 min. Initial conductivity (C1) was measured using an electrical conductivity meter after cooling to 25 °C (Model 335 D, Systronics, Ahmedabad, Gujarat, India). Leaf samples were then placed in a water bath for 10 min at 100℃, cooled to room temperature, and final conductivity (C2) was measured. MSIs were calculated as follows:

MSI = [1 − (C1/C2)] × 100

#### 2.6.3. Assessments of *V. radiata* Growth and Dry Biomass under Varying Levels of Water Stress 

At 50 and 80 DAS, *V. radiata* plants inoculated with drought-tolerant *Enterobacter* sp./*L. adecarboxylata* PAB19 and cultivated in soils at different levels of water stress were detached, germination percentages, root and shoot lengths and dry biomasses, were determined.

#### 2.6.4. Photosynthetic Pigments, Nutrient Uptakes, Symbiosis, and Yields

Leaf pigments (Chl a, Chl b, total chlorophyll, and carotenoid) in the foliage of strain PAB19 inoculated and PEG-treated *V. radiata* were extracted and quantified [38,39] (refer to Section S2.6.4). Nutrient (N and P) uptake by PGPR inoculated and PEG-treated *V. radiata* were analyzed as described by Jackson et al. [40] and Lindner et al. [41], respectively. For estimation of N content, a 10 mL aliquot of digested roots and shoots samples were taken in a 50 mL volumetric flask. To this, 2.0 mL of 2.5 N sodium hydroxide (NaOH) and 1.0 mL of 10% sodium silicate solution were added to neutralize the access of acidity and to prevent turbidity, respectively. The volumetric flask was filled up to the mark of 50 mL by DDW. In a 10 mL graduated test tube, 5.0 mL aliquot of this solution was taken, and 0.5 mL Nessler’s reagent was added allowed to stand for 5 min for color development. The absorbance was read at 525 nm and N content was calculated using the standard curve. For P estimation, one gram of dried samples was digested in nitric acid and perchloric acid prepared in a ratio of 4:1. Then, 5.0 mL of acid digested sample was mixed with 5.0 mL of vanadomolybdate reagent. The volume was made to 25 mL with DDW. The solution was incubated for 30 min until the formation of yellow color. Absorbance was measured at 470 nm.

*Enterobacter* sp./*L. adecarboxylata* inoculated and PEG-treated roots of *V. radiata* were carefully detached and nodules were removed, counted, and oven-dried for 48 h at 80 °C. Dry biomasses of nodules (mg plant^−1^) were then determined. Concentrations of leghemoglobin (LHb) in root nodules were measured quantitatively, as previously described [25]. Seed yields (pod number, pod biomass, seed number, and seed biomass) were also measured. Lowry’s method was used to extract and estimate the grain protein [42].

#### 2.6.5. Effects of *Enterobacter* sp./*L. adecarboxylata* on Stress Biomarkers and Antioxidant-defense Enzymes in *V. radiata* Grown under Conditions of Water Deficit

##### Proline Estimation

Proline contents in leaf tissues of PGPR inoculated and PEG-treated *V*. *radiata* were measured as previously described by [43] (refer to Section S2.6.5.1.1).

##### Estimation of Thiobarbituric Acid Reactive Substances (TBARS)

Malondialdehyde contents in PAB19 inoculated and PEG-treated leaf tissues of *V*. *radiata* were measured as described by [44] (refer to Section S2.6.5.1.2).

#### 2.6.6. Determination of Antioxidant Enzymes

Antioxidant enzyme activity was measured in PGPR inoculated and PEG-treated foliage of *V*. *radiata*. Catalase (CAT), superoxide dismutase (SOD), peroxidase (POD), and glutathione reductase (GR) activities were measured as described by Beers and Sizer [45], Beauchamp and Fridovich [46], and method-1 described by Jablonski and Anderson [47], respectively (refer to Section S2.6.6).

#### 2.6.7. Gas-Exchange Parameters of *V. radiata* Plants Inoculated with PAB19 Strain and Exposed to Varying Levels of Water Stress 

The gas exchange parameters of strain PAB19 inoculated and PEG-treated *V. radiata* foliage were also evaluated. Stomatal conductance (*g*s), rate of transpiration €, internal CO_2_ concentration (*C*_i_), net photosynthetic rate (*P*_N_), and vapor pressure deficit (kPa) were measured using a Li-COR 6400 portable photosynthesis system (Li-COR, Lincoln, NE, USA).

### 2.7. Rhizosphere/Rhizoplane Colonization by Enterobacter sp./L. Adecarboxylata under Water Stress

Rhizosphere and rhizoplane colonization by strain PAB19 were determined as described by Shahid et al. [43] (refer to Section S2.7).

### 2.8. Statistical Analyses

The experiments were performed for two consecutive years under similar experimental conditions and plants were subjected to identical treatments. Each experiment was conducted three times in triplicate. Results are presented as means ± SDs of replicates. Analysis of variance (ANOVA) was performed using the Minitab 17 software package. Dun’an’s Multiple Range Test (DMRT) was used to examine the significance of differences between treatments using a two-way analysis of variance and a significance level of 5%.

## 3. Results and Discussion

### 3.1. Bacterial Variables: Morpho-Biochemical and PGP Traits, Water Stress Tolerance, and 16S rRNA Based Analysis

**Figure 1:**
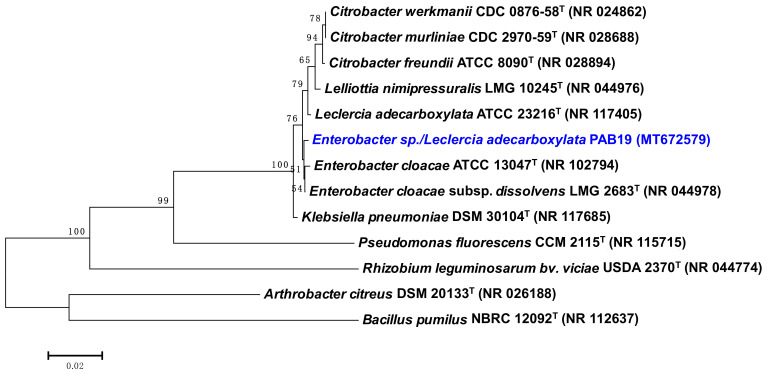
Neighbor-joined phylogenetic tree of *Enterobacter* sp./*Leclercia adecarboxylata* PAB19. The tree was constructed based on 16S rRNA partial gene sequences (taken from NCBI BLAST search tool) from some closely related phylogenetic species (type cultures). Sequences were aligned using the Clustal W sequence alignment tool in MEGA 7.0 software. Bootstrap percentage values as obtained from 1000 replications of the data set are given at nodes. The GenBank accession numbers of isolates and other genera or species are presented in parenthesis. The scale bar corresponds to the mean number of nucleotide substitutions per site.

Globally, drought stress poses a major challenge to agro-sustainability and often results in compromised plant growth, low nutrient uptake, reduced photosynthesis, and disrupted physiological processes. To address this problem, we isolated a drought-tolerant PGPR that could be used as a microbial resource to improve the performance and productivity of crops in water-stressed environments. In this study, a total of 20 rhizospheric isolates were collected and examined for their morphological and biochemical characteristics. All strains were detected under a light microscope as red/pink Gram-negative short rods but each one showing varied responses to biochemical tests. Furthermore, all PGPR isolates were assessed for their efficiency to produce plant growth-promoting substances. Isolates showed a varied level of IAA production when cultured in LB medium supplemented with different concentrations of tryptophan. Generally, the highest production of IAA occurred at 400 µg mL^−1^ of tryptophan. For instance, isolate PAB19 produced the maximum amount of IAA (231 µg mL^−1^ IAA) at 400 µg mL^−1^ of tryptophan (Table 1). Similarly, bacterial isolates showed variable ACC deaminase activity when grown on DF salt medium supplemented with 3.0 mM ACC instead of (NH_4_)_2_SO_4_. Here, bacterial ACC deaminase activity ranged between 13.2 μM α- ketobutyrate mg^−1^ protein hour^−1^ (PAB7) to 29.3 μM α-ketobutyrate mg^−1^ protein hour^−1^ (PAB19). In the liquid culture medium, PAB19 exhibited the maximum solubilization of TCP (68.3 µg mL^−1^). The isolated bacterial strains were checked further to assess the production of NH_3_ and siderophore by growing them in peptone water and nutrient broth medium, respectively. All the isolates showed a positive response to siderophore and ammonia production (Table 1). Among the PGPR isolates, strain PAB19 tolerated an exceptionally higher level of water stress (15% PEG-6000) when cultured on PEG supplemented agar plates (Table 1). Based on its drought tolerance profile, strain PAB19 was selected for inoculation in crop studies. Strain PAB19 exhibited positive reaction for citrate utilization, nitrate reduction, starch, and gelatin hydrolysis, and catalase and peroxidase activity. Based on its biochemical and cultural properties, PAB19 belonged to the genus *Leclercia* and the 16S rRNA gene sequencing identified it at the species level as *Enterobacter* sp./*L. adecarboxylata*. The 16S rRNA nucleotide sequence of strain PAB19 (564 bp) was deposited to GenBank (Accession Number MT672579.1). A similarity search using BLASTn software revealed that strain PAB19 was closely related to *Enterobacter* sp./*L. adecarboxylata* (99.64% sequence identity), which indicated strain PAB19 as *Enterobacter* sp./*L. adecarboxylata*. MEGA 7.0 software was used to create a phylogenetic tree based on a partial 16S rRNA gene sequence (Figure 1) because the 16s rRNA gene sequence analysis is a commonly accepted methodology for bacterial strains identification (at least, at the genus level) [48,49]. 

### 3.2. Plant Growth-Promoting Features of PAB19 under Water Stress Conditions

#### 3.2.1. Production of Indole-3-acetic Acid and Siderophore

PGPR strain PAB19 released many active biomolecules when cultivated in a regulated drought-stressed environment (Table 2). Surprisingly, the PGP activities of strain PAB19 increased as water stress increased. The strain PAB19 synthesized 136.3 ± 6.8 µg mL^−1^ of IAA and this increased with PEG level. For instance, a maximum of 176.2 ± 5.6 µg mL^−1^ of IAA (a 23% increase over the non-PEG-treated control) was recorded at a PEG concentration of 15%. It is estimated that approximately 80% of the rhizospheric bacteria release IAA (a physiologically active auxin) [50], which regulates the growth and various physiological and biochemical processes of plants even under stress (salt, drought, or waterlogging) conditions [51]. Mehmood et al. [52] reported auxin (IAA) promotes root system growth over the long term, which enhances abilities to absorb enough nutrients and water from the soil. In addition, the IAA loosens the plant cell walls leading to enhanced release of root exudates as nutrients for PGPR development [53]. In this regard, various IAA producing drought-tolerant PGPR strains isolated from different ecological niches have been reported to improve the growths of edible crops including legumes. As an example, the inoculation of *Rhodobacter sphaeroides* KE149, an IAA releasing and drought-tolerant bacterium augmented the growth, biomass, chlorophyll content, and nutritional value of adzuki bean plants [23]. Similarly, Danish et al. [48] reported that the drought-tolerant and IAA-producing PGPR strains *P. aeruginosa*, *E. cloacae*, *A. xylosoxidans*, and *L. adecarboxylata* in the presence of biochar increased the overall plant physiology. Additionally, growth, nodulation, physiological traits and water tolerance efficiency of soybean plants were enhanced when inoculated with auxin and gibberellin-secreting and drought tolerating rhizobacterium *P. putida* H-2–3 as reported by Sang-Mo et al. [54].

Under Fe-deficient conditions, siderophores, low molecular weight Fe (iron) chelating compounds produced by some soil microbes, deliver Fe to plants [55]. Here, we have assessed the bacterial production of phenolate siderophore (salicylic acid and 2, 3-dihydroxy benzoic acid) in the presence of a stressor (PEG-6000). Although salicylic acid (SA) is produced by several bacterial genera, when they grow in iron starvation suggesting that it could act as a siderophore, and its production is generally associated with biosynthesis of small ferric-ion-chelating molecules [56], however some studies do not agree with this fact [57]. Like IAA, siderophore production by PAB19 is also elevated at higher PEG levels. Even at a higher level of drought stress (15% PEG), strain PAB19 exhibited the production of siderophore. In the current study, PAB19 synthesized 24.2 ± 1.4 µg mL^−1^ of salicylic acid in the absence of PEG, while 42.5 ± 3.0 µg mL^−1^ was produced at a PEG concentration of 15% (Table 2). Likewise, the maximum amount of 2, 3-DHBA (44.3 ± 2.3 µg mL^−1^) was produced at higher water stress (15% PEG-6000). Microbially produced siderophore usually aids microbial growth in iron-restricted environments. *B. subtilis* strain CAS15 produced a siderophore that protected pepper plants from *Fusarium* wilt (*F. oxysporum* Schl. f.sp. *capsici*) [58], and two siderophore-producing strains of *B. amyloliquefaciens* eliminated *Ralstonia solanacearum*-induced bacterial wilt in tomatoes [59]. Likewise, increased siderophore production at even higher levels of water stress has been reported [60,61].

#### 3.2.2. ACC deaminase Activity

The plant hormone ethylene endogenously regulates plant homeostasis under stress circumstances, resulting in decreased root and shoot development. ACC deaminase-producing bacteria sequester and break down plant ACC to provide N and energy. Furthermore, by eliminating ACC, the bacteria mitigate the negative effects of ethylene, alleviating plant stress and boosting plant development (Glick, 2005). Considering these, we assessed the ACC deaminase activity of *Enterobacter* sp./*L. adecarboxylata* PAB19 in the presence of drought stress. Strain PAB19 cultivated in the medium supplemented with varying levels of PEG showed a good response to ACC deaminase. Increasing PEG concentration significantly increased α-ketobutyrate levels, and a PEG level of 15%, strain PAB19 produced 56.6 ± 5.0 µM α-ketobutyrate mg^−1^ protein h^−1^, which was greater than the amount of 29.3 ± 2.5 ACC deaminase released at a 0% PEG concentration (Table 2). As found in the present study, researchers have reported the production of ACC deaminase by a variety of drought-tolerant soil bacteria subjected to water deficit. In line with the inoculation impact of our culture on *V. radiata*, the exogenous expression of ACC deaminase by drought-tolerant and biofilm-forming *P. azotoformans* FAP5 improved the growth and biochemical attributes of wheat plants raised in soils at different levels of water stress [62]. Similar inoculation impact was reported by Danish et al. [22] where ACC deaminase producing PGPR strains mitigated the adverse effects of drought stress and increased photosynthetic rate, stomatal conductance, and the nutritional value of maize. 

#### 3.2.3. P-solubilizing Activity, HCN, and NH_3_ Production 

Phosphorus is one of the most important plant nutrients and is involved in almost all aspects of plant metabolism, including the synthesis of leaf pigments, respiration, and energy transfer [11]. P scarcity always restricts plant growth and metabolism. However, less than 5% of P in soil is available for plants [63]. Consequently, to prevent P deficiency, phosphatic fertilizers are often used. However, because of their high costs and issues associated with hazardous effluents, plant P supply has shifted toward natural renewable supplies. Phosphate-solubilizing microorganisms (PSM) of several genera also provide alternatives to synthetic P fertilizers [64]. In fact, many drought-tolerant soil bacteria such as *Pseudomonas* [65], *Azospirillum* [66], *Proteus* [67], and *Bacillus* [68] solubilize the soil P under conditions of water stress. In our study, we noticed a similar trend of increased P solubilization activity by strain PAB19 on increasing PEG concentration. The maximum amount (98.3 ± 3.5 µg mL^−1^) of solubilized P was recorded at a high PEG concentration (15%), which represented a 30% increase over the non-treated control (68.3 ± 3.5 µg mL^−1^) (Table 2). Similarly, several drought-tolerant and P-solubilizing PGPR strains viz., *B. amyloliquefaciens* [69] and *Proteus* [67] when inoculated into water-stressed soil systems augment the growth of edible crops. 

However, PEG concentration did not affect the amount of ammonia produced by the PAB19 strain, whereas several researchers have documented the generation of ammonia by drought-tolerant soil bacteria grown in liquid media supplemented with polyethylene glycol [70,71,72].

### 3.3. Effects of Water Stress on Biofilm Development and Associated Traits

**Figure 2:**
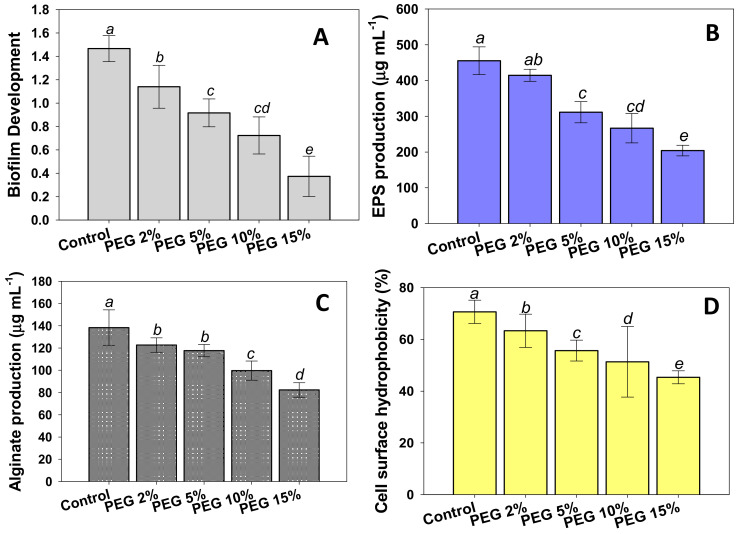
Effects of different levels of water stress (2%, 5%, 10%, and 15% PEG solution) on biofilm development (**A**) and their associated traits; EPS production (**B**), alginate production (**C**), and cell surface hydrophobicity (**D**) of *Enterobacter* sp./*L. adecarboxylata* PAB 19. In these figures, bar diagrams represent the mean values of three replicates. Mean values followed by different letters were significantly different (*p* ≤ 0.05) as determined by the Duncan’s Multiple Range Test (DMRT). Bar and scatter plots represent means ± SDs.

Biofilm development and EPS synthesis are two additional processes that contribute to drought resistance in drought tolerating PGPR. The colonization of plant roots by a rhizobacterium is the very first step for initiating a successful biofilm. In the soil endosphere, rhizoplane, and rhizosphere, there are diverse types of microorganisms. The root-microbe interaction promotes the growth of both plants and PGPR. As a result of a successful plant-microbe interaction, PGPRs successfully colonize the plant root, proliferate into micro colonies, and/or create biofilm [73,74]. Plant-associated biofilms are extremely capable of shielding the host plant from external stress, lowering microbial competition, and providing protective benefits to the host plant, therefore boosting growth, yield, and crop quality. In plant-associated bacterial biofilms, cells and the EPS produced by the cells in response to environmental stimuli work together to sustain the bacterial establishment. Considering these, we decided to evaluate the effect of different levels of water stress on biofilm formation, adhesion of cells to hydrocarbons, and the motility of strain PAB19 in vitro. We found that bacterial biofilm development was dose-dependently reduced in the presence of water stress; however, PAB19 retained the ability to form biofilms even at a PEG concentration of 15% (Figure 2A). 

In addition, we assessed the impact of water stress on EPS production by PAB19. In non-treated controls, PAB19 produced a considerable amount (455.3 µg mL^−1^) of EPS, but it decreased as water stress was increased (Figure 2B). It has been reported that bacterial productions of EPS and alginate tend to improve survival and enhance the productions of active metabolites under harsh conditions such as drought and salinity [75,76]. In our study, the biofilm of PAB19 and associated traits were decreased gradually in a PEG dose-dependent manner, however, strain PAB19 managed to survive and form biofilm and produced the other associated traits. These results show a different trend when compared to the increasing PGP activities of PAB19 under increasing PEG stress (Table 2). This difference could be due to the mode of living of cells of PAB19, i.e., planktonic for PGP activities and static for biofilm formation, as planktonic cells have more contact with the surrounding nutrients and oxygen and are thus able to take up nutrition and release the waste material easily, which is not feasible in the biofilm mode [77,78]. The continuous secretion of EPS by four drought-tolerant PGPR strains in a growth medium supplemented with higher concentrations of PEG was reported by Ghosh et al. [79]. Furthermore, inoculation of exopolysaccharide synthesizing PGPR strains viz. *P*. *aeruginosa*, *Proteus penneri,* and *Alcaligenes faecalis,* enhanced the biomass production, organ lengths, and leaf surface area in maize (*Zea mays* L.) plants, and the soil moisture content [67]. 

Like other biofilm traits, the production of alginate by strain PAB19 was diminished by increasing PEG concentrations. Minimum production (82.3 µg mL^−1^) of alginate was recorded at a PEG concentration of 15% (Figure 2C). Cell surface hydrophobicity (CSH; the adhesion of bacterial cells to hydrocarbons) of strain PAB19 was reduced by increasing the levels of water stress, and PEG at a concentration of 15% maximally reduced CSH by 36% versus non-treated controls (70.6%) (Figure 2D). Bacterial cell surface hydrophobicity is linked to bacterial cell aggregation and adhesion, as well as biofilm formation, and as a result, excessive levels of water stress may impede bacterial colonization behavior [80]. The formation of biofilms and the production of associated traits at high PEG concentrations is a clear indication that the PAB19 strain can withstand severe drought stress, indicating drought tolerance may protect developing bacterial cells and improve survival and activity under water stress.

### 3.4. Plant-Based Studies: Impact of Enterobacter sp./L. adecarboxylata PAB19 Inoculation on V. radiata Grown in Soil Treated with Different Levels of Water Stress under Pot-House Conditions 

#### 3.4.1. Germination Efficiency and Vigor Index 

**Figure 3:**
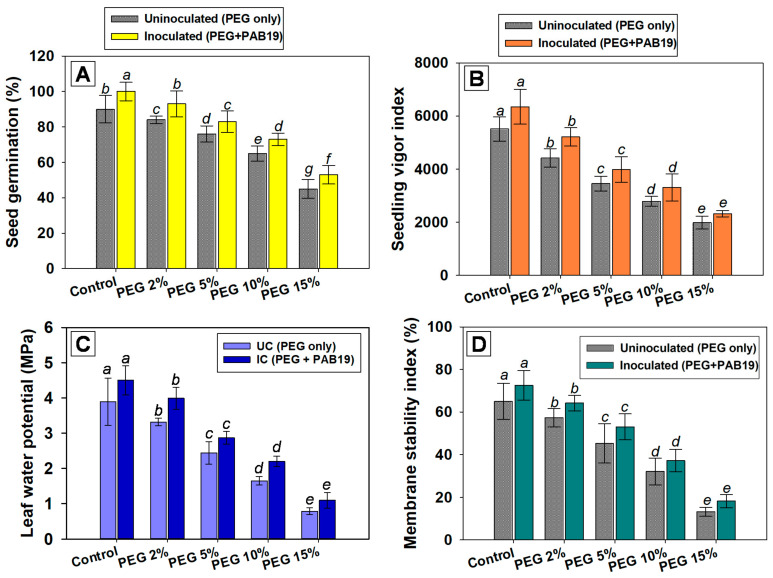
Germination efficiency (**A**), seed vigor indices (**B**), leaf water potential (**C**), and membrane stability indices (**D**) of *V. radiata* (L.) cultivated under different levels of water stress (2%, 5%, 10% and 15% PEG-6000) and inoculated with drought-tolerant *Enterobacter* sp./*L. adecarboxylata* PAB19 under green-house conditions. Bar diagrams and curves represent the mean values of experiments done in triplicate. Mean values followed by different letters are significantly different at *p* ≤ 0.05 as determined by the DMRT. Bar and scatter plots represent means ± SDs.

The efficiency of *V. radiata* seed germination in pot soils amended with different doses of PEG and inoculated with PAB19 was recorded at 6 DAS. Almost all seeds planted in untreated soil germinated, whereas PEG at 15% maximally retarded germination efficiency and SVI by 50 and 56%, respectively, over non-treated controls. However, inoculation of strain PAB19 improved seed germination and the vigor index of *V. radiata* by mitigating water-induced stress (Figure 3A,B). Likewise, the seed germination efficiency of *Astragalus caraganae* grown under water stress was increased following the inoculation of drought-tolerant PGPR strains viz., *A*. *lipoferum*, *A*. *chroococcum*, *P*. *aeruginosa*, and *B*. *cereus* [81]. Additionally, the combined inoculation of drought-tolerant species such as *Mesorhizobium ciceri,* with ACC deaminase positive *Bacillus* sp. and *Pseudomonas* strain 6-P, mitigate the drought stress and simultaneously enhance the growth of *Cicer arietinum* under axenic conditions. It was observed that seedlings with higher osmotic potential (up to 0.4 MPa) had considerably enhanced germination, root and shoot length, and fresh weight compared to non-inoculated control [82].

#### 3.4.2. Effects on Leaf Water Potential and Membrane Stability Index

The leaf water potential and membrane stability index of strain PAB19 inoculated plants cultivated under water deficit were variable. On increasing PEG concentration, ΨL and MSI were significantly (*p* ≤ 0.05) decreased, and 15% PEG solution had the maximum negative effects and reduced ΨL and MSI of *V. radiata* by 79% and 78%, respectively, versus untreated controls. Similarly, Baroowa et al. [83] reported significant reductions in the ΨL and MSI of *V. radiata* under water deficit conditions. However, drought-tolerant *Enterobacter* sp./*L. adecarboxylata* mitigated these toxic effects and improved leaf ΨL and MSI values. For example, in the presence of 2% PEG, PAB19 maximally increased the ΨL and MSI values of *V. radiata* by 18 and 12 %, respectively, versus un-inoculated but 2% PEG-treated plants (Figure 3C,D). These results indicated that inoculation of strain PAB19 had a supporting function in *V. radiata* as the ΨL and MSI of plants were improved under drought-challenged circumstances. It also implies that drought-tolerating strain PAB19 hastened the modification of the plant’s morphology (growth parameters), as well as leaf water potential (physiological properties) probably by synthesizing the IAA.

### 3.5. Growth and Biomass

**Figure 4:**
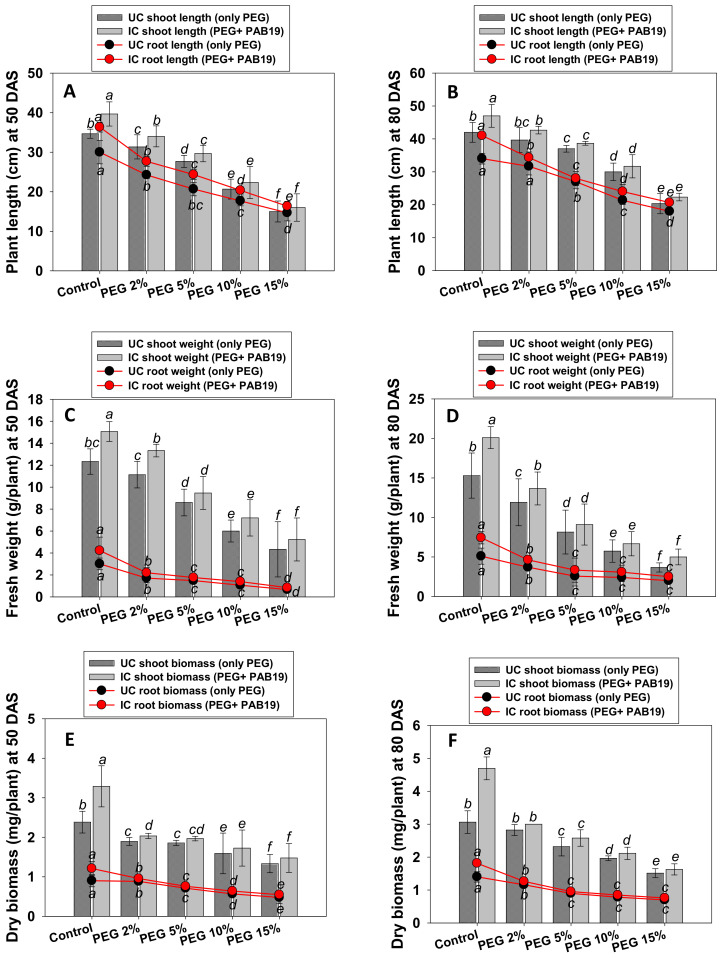
Impact of the bio-inoculation of *Enterobacter* sp./*L. adecarboxylata* PAB19 on biological features; plant length at 50 DAS (**A**) and 80 DAS (**B**), fresh weight at 50 DAS (**C**) and 80 DAS (**D**), dry biomass at 50 DAS (**E**) and 80 DAS (**F**) of *V. radiata* (L.) cultivated under different levels of water stress (2%, 5%, 10% and 15% PEG-6000) under greenhouse conditions. Bar diagrams represent the mean values of three replicates. Mean values followed by different letters are significantly different (*p* ≤ 0.05) as determined by the DMRT. Bar and scatter plots represent means ± SDs.

PGPR bio-primed and PEG supplemented *V. radiata* raised in pots exhibited variable growth (Figure S1). In general, the growth parameters of plants were decreased on increasing PEG concentration, but plant length and weight were enhanced after inoculating PAB19 (Figure 4A–D). For example, lengths and dry biomasses of roots and shoots were severely reduced by 15% PEG. On the other hand, these parameters increased in PAB19 inoculated plants, even under water stress. For example, at 2% PEG, PAB19 maximally increased root and shoots lengths (Figure 4A,B) and dry biomasses (Figure 4E,F) by 13%, 9%, 12%, and 11%, respectively, as compared with un-inoculated, 2% PEG-treated plants. A similar pattern was observed for fresh weights of roots and shoots (Figure 4C,D). The effects of PAB19 and PEG were significant (*p* ≤ 0.05) for all measured parameters. These increases even in presence of water stress were probably due to the enhanced production of IAA and other PGP active chemicals, which aid plants by promoting symbiosis and root morphogenesis in the presence of drought stress. Additionally, it was observed that longer roots were produced after inoculation with *Enterobacter* sp./*L. adecarboxylata* which may aid in the absorption of comparatively more water from deep soil under drought stress circumstances. The strain PAB19 significantly improved the overall performance of *V. radiata*, and we attributed this to the release of growth-regulating substances [84]. IAA, for example, increases root growth, whereas siderophore, ACC deaminase, HCN, and ammonia are involved in plant development. One of the most significant adaptations of plants to withstand the drought is their root system design. The treatment of drought tolerating PGPR has been shown to increase root development and change root architecture in plants. It has also been suggested that bacterial-induced changes in root architecture might contribute to an increase in total root surface area, therefore enhanced water and nutrient absorption, with favorable consequences for overall plant development [18]. Similarly, several drought-tolerant PGPR strains have been reported to increase the growth and dry biomasses of edible crops, including legumes, cultivated under water stress [85,86]. For instance, Naseem and Bano (2014) used strain *Alcaligenes faecalis* AF3 with *Zea mays* seedlings in growth chamber experiments [67]. In that study, drought challenged but PGPR treated plants had a 10% increase in root length three weeks after planting over drought-stressed but non-inoculated control plants. They claimed that PGPR treatment caused an increase in length and biomass of root resulting in an overall increase in water absorption, allowing treated plants to withstand the drought.

### 3.6. Leaf Pigments and Nutrient Uptake

**Figure 5:**
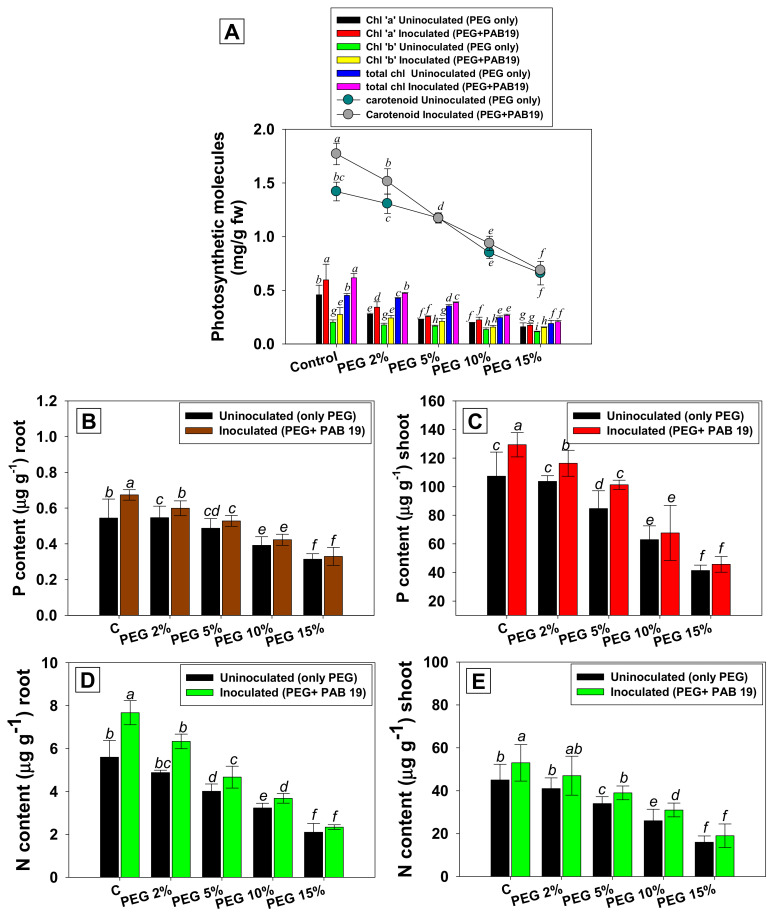
Impacts of the bio-inoculation of *Enterobacter* sp./*L. adecarboxylata* PAB19 on leaf pigments (**A**), shoot P (**B**), root P (**C**), shoot N (**D**), and root N (**E**) of *V. radiata* (L.) cultivated under different levels of drought stress (2%, 5%, 10% and 15% PEG-6000) under greenhouse conditions. Mean values followed by different letters are significantly different (*p* ≤ 0.05) as determined by the DMRT test. Bar and scatter plots represent means ± SDs. Bar and scatter plots represent means ± SDs.

Photosynthesis is a crucial physicochemical process in higher plants and has a direct influence on biomass production; it is particularly vulnerable to drought. Leaf pigments and symbiotic features of *Enterobacter* sp./*L. adecarboxylata* PAB19 and PEG-treated *V*. *radiata* plants decreased on increasing water stress. In the current study, drought stress impacts plant photosynthetic properties, which in turn influences the physiological and biochemical processes, at the whole-plant level. For example, PEG-6000 solution at 15% greatly diminished chl a, chl b, total chlorophyll, and carotenoid contents by 64%, 45%, 57%, and 53%, respectively, versus untreated controls. It suggests the loss of photosynthetic pigments by high levels of drought stress that could be due to the increased activity of certain drought-induced enzymes reducing the production of photosynthetic pigments under drought stress. However, PAB19 inoculation of plants raised in soil supplemented with 2% PEG maximally increased chl-a, chl-b, total chlorophyll, and carotenoid contents by 21%, 29%, 12%, and 16%, respectively (Figure 5A). The data obtained in the present study suggest that under drought stress, strain PAB19 directly regulates the plant physiology by synthesizing plant hormone (IAA), as well as by improving the mineral and nitrogen availability. The increased photosynthetic pigments by drought-tolerant PGPR in the current study may probably be connected to the development of chloroplast. A similar increase in the formation of photosynthetic pigments in maize plants raised at various levels of water stress was observed after inoculating drought-tolerant PGPR strains, namely, *P*. *aeruginosa*, *E. cloacae,* and *L. adecarboxylata* [48]. Furthermore, inoculations of *B*. *pumilus* and *Pseudomonas* sp. increased water stress tolerance and increased the chlorophyll levels in maize cultivated under conditions of water deficit [87]. 

The N and P accumulations in the tissues of PAB19 inoculated and PEG-treated *V*. *radiata* plants were variable. On increasing water stress, amounts of N and P fell significantly (*p* ≤ 0.05). However, after inoculating PAB19, the nutritional contents of *V. radiata* significantly improved. For example, maximum increases in root P, shoot P, root N, and shoot N of 10%, 11%, 23%, and 13% were recorded when plants were grown in soil treated with 2% PEG and inoculated with PAB19 (Figure 5B–E). In brief, the analysis showed that inoculation of drought-tolerant PGPR and PEG amended *V. radiata* plants had considerably higher nutrient content (P and N), as compared to controls under water deficit conditions, allowing higher plant performance. This increment in nutrient uptake by plants might be due to the mobilization of various nutrients from the soil to plant root surfaces by soil bacterial activity. Both non-inoculated and PAB19 inoculated plants tend to accumulate more nitrogen in their shoots when subjected to a water shortage. Plant softening increases the concentration of N in leaves to continue growth in dry conditions. The P content was greater in un-inoculated and PAB19 inoculated *V. radiata* shoots as compared to the P content in roots. A similar trend was followed by N. This might be linked to plant’s capacity to balance the distribution of nutrients across their compartments as a strategy for responding to environmental changes [88]. In a similar study, improved root growth and enhanced nutrient use efficiency by *Zea mays* plants grown in soil inoculated with drought-tolerant *P*. *fluorescens* S3X or *Cupriavidus necator* 1C2 under water deficit conditions has been reported [89]. In addition, Adesemoye et al. (2008) found that drought-tolerant microbial inoculants improved maize growth as well as N and P absorption efficiency [90]. Recently, it was demonstrated that inoculation of beneficial soil bacteria viz., *Staphylococcus sciuri*, *Zobellella denitrificans*, and *Arthrobacter endophyticus* caused an upsurge in nutrient uptake of plants raised in soil showing different levels of drought stress [91]. 

### 3.7. Effect of Enterobacter sp./L. adecarboxylata on Symbiosis and Yield Attributes

#### 3.7.1. Nodule Numbers, Biomass, and LHb Contents

**Figure 6:**
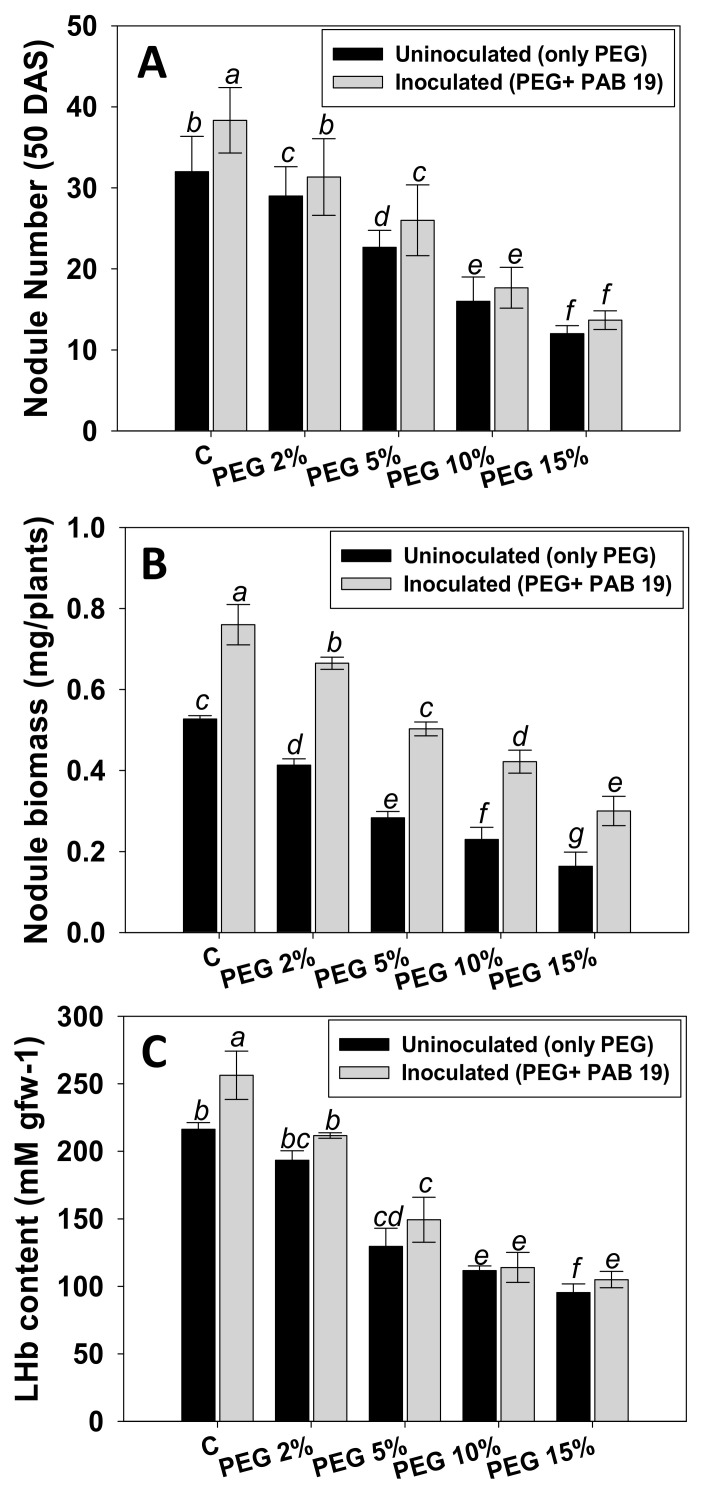
Impact of *Enterobacter* sp./*L. adecarboxylata* PAB19 on symbiotic features such as nodule number (**A**), nodule biomass (**B**), and LHb content (**C**) at 50 DAS of *V. radiata* (L.) in the presence of different levels of water stress (2%, 5%, 10% and 15% PEG-6000) under greenhouse conditions. Bar diagrams represent the mean values of three replicates. Mean values followed by different letters are significantly different (*p* ≤ 0.05) as determined by the DMRT. Bar and scatter plots represent means± SDs.

Nodule numbers, dry biomass, and LHb contents of *V. radiata* were maximally reduced by 62%, 69%, and 56%, respectively, in the presence of 15% PEG at 50 DAS (Figure 6). However, inoculation of PAB19 considerably increased these symbiotic features of plants cultivated in water-stressed soils. For example, PAB19 greatly enhanced nodule numbers, biomass, and LHb content by 10%, 38%, and 9%, respectively, in the presence of a 2% PEG at 50 DAS (Figure 6A–C). Nodule number was found to be strongly correlated with nodule biomass LHb content (R^2^ = 0.89 and 0.85, respectively). The data suggest that PEG-induced decrease in symbiotic efficiency (number, biomass, and LHb content of nodule) of *V. radiata* was reversed by increased nodulation following the inoculation of drought tolerating, IAA-synthesizing, and other bio-molecule-producing PAB19. It is also suggested that through both local and systemic hormone signaling, successful deployment of bacteria in the *V. radiata* rhizosphere enhanced plants’ symbiotic attributes. Similarly, in a previous study, drought-tolerant PGPR strains recovered from the root nodules of mung bean improved nodulation, and the symbiotic features of *V. radiata* raised under abiotic stress conditions [92]. Corroborating our results, inoculation of *Pisum sativum* (pea) plants growing in dried soils with ACC deaminase producing PGPR strain *Variovorax paradoxus* 5C-2 improved the plant growth [93]. They claimed that bacterized plants showed more seed yield, seed number, and seed nitrogen accumulation and restored nodulation which was repressed in drought stress conditions. Co-inoculation of *P. vulgaris* L. (common bean) with drought tolerating PGPR strains (*Rhizobium tropici* CIAT 899, *P. polymyxa* DSM36, and *P. polymyxa* Loutit), resulted in higher growth and nodulation relative to plant raised in soil treated only with drought stressor (PEG) [94].

#### 3.7.2. Seed Yields 

Seed features of *V*. *radiata* cultivated in soils at different levels of water deficit exhibited substantial decreases. Maximum decreases in seed parameters were observed at a PEG level of 15%. When PAB19 was administered to *V. radiata* growing in soil treated with 2% PEG, pod numbers, yield, seed numbers, seed biomass, and grain protein content rose maximally (*p* ≤ 0.05) by 20%, 12%, 11%, 10%, and 10%, respectively (Figure S2A–E). PAB19 and PEG had a significant synergistic effect (bio-inoculum × PEG) on the yield attributes of *V. radiata*. Plant adaptation is connected with high concentrations of solutes such as soluble carbohydrates and protein to control the osmotic potential of cells, which, in turn, induces an improvement in water absorption under adverse conditions to withstand drought stress. Similarly, drought stress had no influence on growth, seed yield, and ripening of *P. sativum* in both pot and field studies when ACC deaminase-producing *Pseudomonas* spp. was used [95]. Furthermore, the application of drought-tolerant PGPR strain *V. paradoxus* 5C-2 to *P*. *sativum* plants caused an improved growth, seed yield, and water-use efficiency [96]. Likewise, it has been reported that drought tolerating PGPR strains improved the seed features of wheat crops raised in a water-stressed soil system [97].

### 3.8. Impacts of Enterobacter sp./L. adecarboxylata Bio-Inoculation on Stress Markers and Antioxidant Enzymes in V. radiata at Different Levels of Water Stress 

#### 3.8.1. Proline and MDA Contents

**Figure 7:**
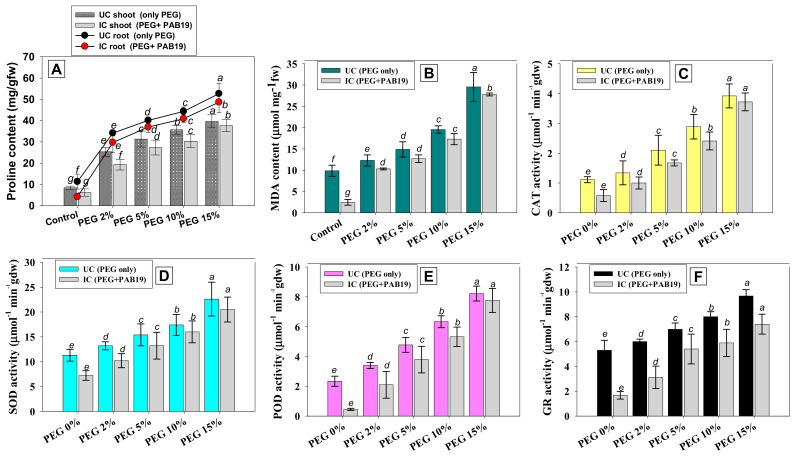
Impact of drought-tolerant *Enterobacter* sp./*L. adecarboxylata* PAB19 on stress-related parameters; proline (**A**) and MDA (**B**) contents and antioxidant enzyme activities; CAT (**C**), SOD (**D**), POD (**E**), and GR (**F**) of *V. radiata* cultivated in the presence of different levels of water stress (2%, 5%, 10% and 15% PEG-6000) raised under greenhouse conditions. Bar diagrams represent the mean values of three replicates. Mean values followed by different letters are significantly different (*p* ≤ 0.05) as determined by the DMRT. Bar and scatter plots represent means ± SDs.

Stress-induced physiological and functional activities can be defined based on accumulations of stressor chemicals (e.g., proline) in plant tissues. In plants, proline (that often acts as an osmolyte) is generated in response to drought stress [98]. Proline synthesis is a frequent response in drought-stressed crops, and it serves to preserve the cell membrane and macromolecule structure throughout stressful conditions. The buildup of proline under stress offers energy for growth and survival, as well as assisting crops in coping with abiotic stressors. Under water stress, proline acts as an osmolyte, accumulating faster and assisting plants in maintaining cell turgor potential [98]. In many plant species, elevated levels of free cellular proteins protect against various biotic and abiotic stressors [99]. In the present study, proline and MDA accumulated in a dose-dependent manner within the fresh foliage of PEG-treated and PAB19 inoculated *V. radiata*, and at a PEG concentration of 15%, shoot proline was maximally induced at 39.6 µg g^−1^ fw (78% higher versus non-treated controls) and shoot MDA was maximally induced at 29.5 µg g^−1^ fw (67% higher than the control) (Figure 7A,B). However, the presence of drought-tolerant strain PAB19 declined the water stress-induced oxidative stress in *V. radiata*. For example, PAB19 significantly (*p* ≤ 0.05) and maximally lowered shoot proline and MDA levels by 25% and 20%, respectively, when applied to *V*. *radiata* plants cultivated in soils containing PEG at 2%. Similarly, two drought-tolerant PGPR strains *Bacillus cereus* P2 and *Planomicrobium chinense* P1 reduced proline and malondialdehyde contents when inoculated to *Helianthus annus* under water-stressed conditions [100], and decreases in proline and MDA contents in foliage were observed in water-stressed chickpea plants inoculated with three drought-tolerant PGPR strains, namely, *B. subtilis*, *B. thuringiensis,* and *B. megaterium* [101]. Similarly, three drought-tolerant PGPR, *P. jessenii*, *P. synxantha* and *A. nitroguajacolicus* significantly improved the plant growth by modulating the stress-related enzymes, lowering the level of H_2_O_2_, and malondialdehyde (MDA) content in plants under drought stress relative to non-inoculated plants [102]. These findings support the use of PGPR to improve plant drought tolerance by changing antioxidant activity and stressor metabolites (proline and MDA) in water-stressed situations.

#### 3.8.2. Antioxidative Defense Enzymes

Plants have antioxidant defense mechanisms to protect themselves from the damaging effects of oxidative stress. We noticed that antioxidant enzyme (AE) activity was increased in the foliage tissues of *V. radiata* on increasing PEG concentrations. PEG had the greatest impact at 15% on AE activity and increased CAT, SOD, POD, and GR levels by 72%, 50%, 84%, and 45%, respectively, over untreated controls (Figure 7, panel C–F). However, PAB19 inoculation mitigated the negative effect of water stress by lowering AE levels in foliage tissues. For example, at a PEG concentration of 2%, PAB19 significantly reduced CAT, SOD, POD, and GR levels by 25%, 22%, 38%, and 48%, respectively, versus un-inoculated but 2% PEG-treated *V. radiata* (Figure 7C–F). Observed reductions in antioxidant enzyme levels in *Enterobacter* sp./*L. adecarboxylata* bio-primed plants cultivated in soils containing various levels of PEG may be linked to reduced water deficit, and thus, to less oxidative damage. Here, inoculation of PAB19 reduced the synthesis of these ROS-scavenging enzymes by *V. radiata* plants that showed reduction in oxidative stress. Experiments on bacterial-mediated tolerance have evaluated antioxidant enzyme activity to determine the role of the scavenging system under drought stress. This research specifically looked at whether the treatment of drought tolerating PGPR to plants resulted in decreased levels of antioxidant enzymes. In this context, it was discovered that *Bacillus subtilis* EPB and *Pseudomonas fluorescens* Pf1 modulated the activity of CAT in *V. radiata* plants [103]. The changes were shown to be connected to the observed drought tolerance. Additionally, drought-tolerant *Ochrobactrum* sp. BRISH6 mitigates the toxic effect of deficit water stress and lowered levels of components of the antioxidative defense system, viz., SOD, CAT, APX, POD, GPX, and polyphenol oxidase, in *Zea mays* [20]. Similarly, PGPR strain *P. pseudoalcaligenes* alleviated water stress in *Z. mays* and enhanced antioxidant defense enzyme levels by modulating the defense system under different levels of water stress [21]. Likewise, plants treated with drought tolerating strains of *Bacillus* species acquired drought resistance by lowering the activity of antioxidant enzymes such as APX and GR [71].

### 3.9. Effect of Enterobacter sp./L. adecarboxylata on the Gas Exchange Parameters of V. radiata under Drought Stress 

**Figure 8:**
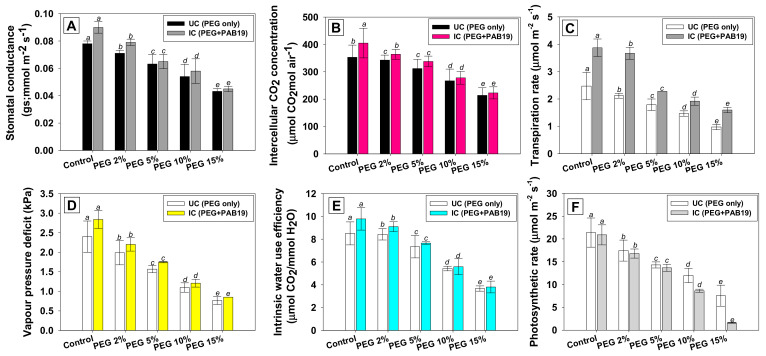
Impact of *Enterobacter* sp./*L. adecarboxylata* PAB19 on gas exchange parameters; stomatal conductance (**A**), intercellular CO_2_ concentration (**B**), transpiration rate (**C**), vapor pressure deficit (**D**), intrinsic water use efficiency (**E**), and photosynthetic rate (**F**) of *V. radiata* cultivated in the presence of different levels of water stress under green-house conditions. Bar and scatter plots represent means± SDs. Mean values followed by different letters are significantly different (*p* ≤ 0.05) as determined by the DMRT.

Physiological characteristics (stomatal conductance, CO_2_ concentration, transpiration rate, vapor pressure deficit, intrinsic water use efficiency, and photosynthetic rate) are useful tools for studying the effects of drought stress on a variety of plants. The leaf gas exchange parameters of PEG-treated and strain PAB19 inoculated *V. radiata* plants were evaluated. Similar trends were observed for gas exchange attributes, that is, stomatal conductance (*g*s), intercellular CO_2_ concentration (*C*i), rate of transpiration (*E*), vapor pressure deficit (VpDL), intrinsic water use efficiency (iWUE), and photosynthetic rate (*P*N). Under drought conditions, these parameters of *V. radiata* seedlings were significantly (*p* ≤ 0.05) and markedly reduced at 15% PEG-6000 versus untreated controls. However, seedlings inoculated with PAB19 showed greatest improvement in these parameters. For example, *g*s, *C*i, *E*, VpDL, iWUE, and *P*N were significantly (*p* ≤ 0.05) increased by 12%, 10%, 43%, 13%, 12%, and 9% in PAB19 inoculated *V. radiata* plants raised in soil amended with 2% PEG (Figure 8A–F), which shows PAB19 improves these activities by reducing the impacts of drought stress, which may be due to its high resilience and effective colonization of the rhizosphere. Furthermore, the changes in CO_2_ assimilation for photosynthesis might result from these improvements in physiological characteristics. Our findings demonstrate that drought tolerating strain PAB19 not only boosts the water and nutrient absorption, but also improves the stomatal conductance, which helps to buffer the detrimental effects of drought. *V. radiata* plants inoculated with strain PAB19 exhibited lower levels of potentials and higher water content in response to drought stress, allowing inoculated and amended plants to maintain high organ hydration and turgor levels, which support overall physiological activities of the cells, particularly those linked to the photosynthetic apparatus. Similarly, inoculation of *Platycladus orientalis* seedlings with cytokinin-producing *Bacillus subtilis* enhanced ABA levels in shoots and stomatal conductance, giving drought stress tolerance [104].

### 3.10. Rhizosphere and Rhizoplane Colonization under Water Deficit Conditions

Root colonization is an important component of plant/microbe interactions in the rhizosphere. Plant growth and production, and protection from biotic and abiotic factors, may benefit from this symbiotic relationship [105]. PEG untreated but strain PAB19 treated roots exhibited bacterial colonization, whereas the extent of colonization was found less on PEG-treated roots. Bacterial components such as EPS, cell wall polysaccharides, and extracellular bacterial proteins may sustain root surface attachment. At 50 and 80 DAS, rhizosphere and rhizoplane population numbers of strain PAB19 differed. Furthermore, CFU counts of PAB19 were considerably reduced when PEG concentrations were increased. However, PAB19 was able to survive and colonize even at the maximum dose of PEG (15%); lower PEG doses had comparatively less effect on bacterial population viability counts. Rhizospheric CFU counts of PAB19 were 2.33 and 2.11 log CFU g^−1^ after treatment with 15% PEG solution and 8.46 and 6.67 log CFU g^−1^ for untreated controls at 40 and 80 DAS, respectively (Figure S3). Similar trends were observed for rhizoplane counts. As PGPR colonize root surfaces, they proliferate and reproduce by absorbing major signaling chemicals and nutrients from root exudates and form biofilms in the root system. Likewise, drought tolerating PGPR strains viz., *Flavobacterium* sp., *E. ludwigi,* and *Klebsiella* sp., successfully colonized the surface and interior roots of wheat plants even under drought stress as was observed by scanning microscopy [106]. Colonization may have triggered numerous physiological mechanisms that assisted plants in maintaining photosynthesis and plant development under drought stress.

In this study, we have reported the PGP aspect of *Enterobacter* sp./*L. adecarboxylata* PAB19 under drought stress. Its pathogenicity cannot be judged in humans since the response of strain PAB19 as an opportunistic pathogen was not studied. However, studies have reported *Enterobacter* sp./*L. adecarboxylata* to cause infection in immunocompromised patients. Many of the PGPR strains lack classical pathogenicity genes and the pathogenicity could be a strain specific feature. For example, *Burkholderia cepacia* and members of the *Burkholderia cepacia* complex (BCC) have pathogenicity-related genes and given a particular host, have the opportunity to colonize it [107]. Other members of the same genus lack these elements and are the ones that have potential for use as PGPR [108,109]. Origin of isolation of a specific strain may also determine its nature as opportunistic human pathogen. For instance, strains of *Bacillus subtilis*, *B. thuringiensis*, and *Saccharomyces cerevisiae* could be opportunistic pathogens [110,111], however, many other strains of these species are widely used as PGPR, biopesticide, in wine and bread industries [112,113,114]. Further studies are warranted to check the potential pathogenicity of the strain PAB19 before utilizing it as a field inoculant.

## 4. Conclusions

In the scope of sustainable agriculture and environmental health, novel solutions for not only enhancing stress tolerance and crop yields but also lowering farmers’ reliance on agrochemicals need to be researched and adopted. We have shown that water deficiency detrimentally affects germination efficiency, biological features, symbiosis, seed characteristics, and nutrient uptake by *V. radiata*. Additionally, leaf exchange parameters were increased and stress biomarkers, antioxidants, and antioxidant enzymes were induced by PEG in *V. radiata*. As an organic strategy for *V. radiata* cultivation, a drought-tolerant PGPR strain of *Enterobacter* sp./*L. adecarboxylata* PAB19 capable of withstanding high PEG-6000 concentrations while secreting the plant growth-regulating substances circumvented the drought stress in our experimental conditions. PAB19 inoculation of *V. radiata* protected it from drought stress and enhanced the biological attributes viz., leaf pigments, symbiosis, and yield attributes, and uptake of essential nutrients (N and P) were simultaneously enhanced. The improvement due to PAB19 inoculation-induced mitigation of stress was a result of the release of active PGP substances following rhizosphere and rhizoplane colonization by PAB19. Additionally, our findings suggest that strain PAB19 develops drought-adaptive strategies and affects some of the essential plant processes such as photosynthesis, antioxidant enzyme production, leaf-exchange characteristics, and decreased lipid peroxidation. To summarize, the drought-tolerant PGPR strain *Enterobacter* sp./*L. adecarboxylata* PAB19 offers an appealing, agronomically viable, and long-term potential alternative for *V. radiata* cultivation even under drought conditions.

## Figures and Tables

**Table 1:** Plant growth-promoting features of the PGPR isolates (PAB1 to PAB20) recovered from rhizosphere soils and their tolerance to water stress (PEG-6000).

Bacterial Isolates	IAA Production (µg mL^−1^)					ACC Deaminase (μmol α-KB/mg Protein/h)	P-Solubilization (µg mL^−1^)	NH_3_ Production	Siderophore (FeCl_3_ test)	Water Stress Tolerance to PEG-6000
	0T	100T	200T	300T	400T					
PAB1	23.2	27.2	36.7	47.1	67.2	17.5	43.7	++	++	5%
PAB2	14.3	18.0	24.3	29.5	36.1	19.3	37.8	+	++	2%
PAB3	9.23	15.7	22.6	35.4	42.7	22.0	34.2	+	++	7%
PAB4	11.0	18.1	26.3	37.9	52.0	21.2	45.7	+	++	8%
PAB5	26.8	43.2	67.6	73.2	87.1	17.4	49.0	++	++	5%
PAB6	8.9	16.8	28.7	40.1	55.0	18.4	52.5	++	++	9%
PAB7	10.8	17.4	26.0	41.4	52.7	13.2	34.1	++	++	10%
PAB8	23.7	32.8	46.8	65.9	87.4	19.3	30.9	++	++	12%
PAB9	14.3	19.3	32.1	42.5	53.1	20.3	46.2	++	++	5%
PAB10	2.6	4.8	17.3	22.7	31.2	22.5	57.3	++	++	7%
PAB11	13.2	18.0	24.6	32.1	45.7	24.2	38.2	+	++	3%
PAB12	21.2	36.5	48.6	63.4	87.3	25.6	23.9	+	++	7%
PAB13	12.0	16.7	28.4	35.6	43.0	23.4	28.9	++	++	5%
PAB14	34.3	44.2	56.7	89.4	112.0	25.6	55.4	+	++	5%
PAB15	7.8	11.9	18.5	26.4	32.4	28.1	61.7	+	++	8%
PAB16	14.3	28.0	43.2	56.3	70.2	23.4	38.6	+	++	10%
PAB17	11.8	16.3	23.1	32.0	47.2	28.4	43.2	++	++	12%
PAB18	6.7	17.4	22.4	28.5	38.9	24.6	56.0	++	++	10%
PAB19	89.4	136.3	165	189	231	29.3	68.3	++	++	18%
PAB20	21.2	39.0	75.3	84.3	92.1	25.0	49.0	++	++	7%

IAA = indole-3-acetic acid; α-KB = α-keto butyrate; T = tryptophan; Single ‘+’ denotes moderate production while ‘++’ represents high production.

**Table 2:** Quantification of PGP substances or activity of *Enterobacter* sp./*L. adecarboxylata* PAB19 grown with 2–15% PEG-6000 stress.

Treatment	Dose Rate (%)	IAA Production (µg mL^−1^)	ACC Deaminase (μmol α-KB/mg Protein/h)	P-Solubilization (µg mL^−1^)	Production of Siderophore Production				
					HCN	NH_3_	FeCl_3_ Test	SA (µg mL^−1^)	2,3 DHBA (µg mL^−1^)
**Drought** **(PEG-6000)**	0	136.3 ^d^ ± 6.8	29.3 ^d^ ± 2.5	68.3 ^e^ ± 3.5	−	+++	++	24.2 ^e^ ± 1.4	34.1 ^d^ ± 0.5
	2	139 ^d^ ± 10.3	31.0 ^d^ ± 7.0	74.6 ^d^ ± 3.0	−	++	++	27.3 ^d^ ± 2.1	36.0 ^c^ ± 1.1
	5	157.6 ^c^ ± 8.5	37.0 ^c^ ± 2.0	81.6 ^c^± 3.2	−	++	++	31.5 ^c^ ± 2.5	38.5 ^b^ ± 1.7
	10	169.6 ^b^ ± 5.5	44.0 ^b^ ± 2.0	91.6 ^b^ ± 3.5	−	++	++	37.0 ^b^ ± 2.8	40.0 ^b^ ± 1.8
	15	176.2 ^a^ ± 5.6	56.6 ^a^ ± 5.0	98.3 ^a^ ± 3.5	−	+	++	42.5 ^a^ ± 3.0	44.3 ^a^ ± 2.3

IAA = indole-3-acetic acid; α-KB = α-keto butyrate; HCN = hydrogen cyanide; SA = salicylic acid; 2,3 DHBA = 2, 3-dihydroxybenzoic acid. ‘+’ and ‘–‘ represent positive and negative reactions, respectively. Values are the mean of three independent replicates. Mean values denoted with different letters are significantly different (*p* ≤ 0.05) as determined by the DMRT test. Values represent means ± SDs. ‘+’, ‘++’, and ‘+++’ denote low, moderate, and high production, respectively.

## Data Availability

The data presented in this study are available in the main manuscript and in the supplementary material here.

## Supplementary Materials

The following are available online at https://www.mdpi.com/article/10.3390/biology10111149/s1, Figure S1. Representative pictures of bio-inoculated V. radiata plants with drought-tolerating *Enterobacter* sp./*L. adecarboxylata* PAB19 growing with 2%, 5%, 10%, and 15% PEG solution in the soil., Figure S2. Impacts of PAB19 on yield parameters; pod number (A), pod yield (B), seed number (C), seed yield (D), and protein content (E) of V. radiata cultivated in the presence of different levels of water stress (2%, 5%, 10% and 15% PEG-6000) under green-house conditions. Bar diagrams represent the mean values of three replicates. Mean values fol-lowed by different letters are significantly different (*p* ≤ 0.05) as determined by the DMRT test, Figure S3. Colonization of the root surfaces of V. radiata by *Enterobacter* sp./*L. adecarboxylata* strain PAB19. Rhizoplane and rhizosphere colonization in the presence of different levels of PEG at two different seeding stages (50 and 80 DAS). Mean values followed by different letters are significantly different (*p* ≤ 0.05) as determined by the DMRT.
