# Supplementary material for: Drought Tolerant Enterobacter sp./Leclercia adecarboxylata Secretes Indole-3-acetic Acid and Other Biomolecules and Enhances the Biological Attributes of Vigna radiata (L.) R. Wilczek in Water Deficit Conditions
Source: Biology (Basel). 2021 Nov 8;10(11):1149. doi: 10.3390/biology10111149 (PMC8614786; doi:10.3390/biology10111149)
Supplement: Supplementary file 1 [file biology-10-01149-s001.zip › biology-1382409-supplementary.pdf]

biology

MDPI

Drought Tolerant *Enterobacter sp./Lecrercia adecarboxylata* secretes Indole-3-Acetic Acid and other Biomolecules and Enhances the Biological Attributes of *Vigna radiata* (L.) R. Wilczek in Water Deficit Conditions

Bilal Ahmed [1,*], Mohammad Shahid [2], Asad Syed [3], Vishnu D. Rajput [4], Abdallah M. Elgorban [3], Tatiana Minkina [4], Ali H. Bahkali [3] and Jintae Lee [1,*]

[1] School of Chemical Engineering, Yeungnam University, Gyeongsan, 38541, Republic of Korea
[2] Department of Agricultural Microbiology, Faculty of Agricultural Sciences, Aligarh Muslim University, Aligarh 202002, India; gd4858@myamu.ac.in
[3] Department of Botany and Microbiology, College of Science, King Saud University, P.O. 2455, Riyadh 11451, Saudi Arabia; assyed@ksu.edu.sa (A.S.), aelgorban@ksu.edu.sa (A.M.E.), abahkali@ksu.edu.sa (A.H.B.)
[4] Academy of Biology and Biotechnology, Southern Federal University, Rostov-on-Don-344090, Russia; rvishnu@sfedu.ru (V.D.R.), tminkina@mail.ru (T.M.)
* Correspondence: bilal22000858@yu.ac.kr (B.A.); jtlee@ynu.ac.kr (J.L.)

Citation: Ahmed, B.; Shahid, M.; Syed, A ; Rajput, V D. ; Elgorban, A M. ; Minkina, T ; Bahkali, A ; Lee, J . Drought Tolerant Enterobacter sp./Leclercia adecarboxylata secretes Indole-3-Acetic Acid and other Biomolecules and En-hances the Biological Attributes of Vigna radiata (L.) R. Wilczek in Water Deficit Conditions. *Biology* **2021**, *10*, 1149. https://doi.org/10.3390/biology10111149

Academic Editor: Ana Alexandre and Kathrin Wippel

Received: 31 August 2021
Accepted: 1 November 2021
Published: 5 November 2021

Publisher's Note: MDPI stays neutral with regard to jurisdictional claims in published maps and institutional affiliations.

Copyright: © 2021 by the authors. Licensee MDPI, Basel, Switzerland. This article is an open access article distributed under the terms and conditions of the Creative Commons Attribution (CC BY) license (http://creativecommons.org/licenses/by/4.0/).

S2.3 16S rRNA partial gene sequencing of PAB19

The bacterial isolate PAB19 was grown overnight in nutrient broth at 28ºC. Subsequently, genomic DNA was extracted and the target gene was amplified by PCR. For molecular characterization, the 16S rRNA gene was amplified by PCR using universal primers 785F (5'-GGA TTA GAT ACC CTG GTA -3') and 907R (5'-CCG TCA ATTCMTTT RAG TTT-3'). Amplification conditions were as follows: (i) 94 °C for 5 min, (ii) 35 cycles of 94°C for 30 s (denaturation), (iii) 55.6°C for 45 s (annealing), (iv) 72°C for 1.5 min (elongation), and (v) a final elongation step at 72°C for 10 min. Amplicon production was confirmed by running 1.2% agarose gel electrophoresis and visualizing the product using ethidium bromide (EtBr) and captured in the Gel Documentation system (Gel Doc™ XRS + Imaging System). The 16S rRNA gene sequence analysis of the PCR amplified product was done by Macrogen Inc. (Seoul, South Korea) using the online tool BLASTn (https://blast.ncbi.nlm.nih.gov/Blast.cgi?PAGE_TYPE=BlastSearch). The sequences for tree preparation were downloaded in FASTA format, aligned by Clustal W in MEGA 7.0 and a phylogenetic tree was constructed using the Neighbour-joining method in MEGA 7.0 (https://www.megasoftware.net).

S2.4 Plant growth regulating (PGR) substances released by Enterobacter sp./L. adecarboxylata PAB19 under different levels of water stress

S2.4.2 Bioassay of siderophore production

The PGPR isolates were spot inoculated on 0-15% PEG supplemented universal chrome azurol S (CAS) agar plates and incubated at 28±2 °C to produce an orange color zone (halo) around bacterial colonies. Furthermore, siderophore levels were quantitatively evaluated by growing bacteria in PEG amended iron (Fe) free succinate liquid medium, as suggested by Barbhaiya and Rao [1]. Siderophore production was estimated using the universal chrome azurol liquid assay [2]. Siderophore units were calculated as follows:

$$\% \text{ Siderophore unit} = \frac{\lambda \text{ of reference (Ar)} - \lambda \text{ of test (As)}}{\lambda \text{ of reference (Ar)}} \times 100$$

where Ar = Absorbance of un-inoculated media and CAS solution

As = Absorbance of the test sample (culture supernatant and CAS solution).

By cultivating the *Enterobacter sp./L. adecarboxylata* PAB19 in Modi medium (K_2HPO_4 0.05%; $MgSO_4$ 0.04%; NaCl 0.01%; mannitol 1.0%; glutamine 0.1%; NH_4NO_3 0.1%), the

production of phenolate siderophore (SA and 2, 3-DHBA) was quantified. Bacterial cells were added to Modi medium and cultured for 5 days. After incubation, the culture broth was centrifuged at 8000 rpm, and the concentrations of catechol-type phenolates salicylic acid and 2, 3-dihydroxybenzoic acid in the supernatant were measured using a version of Hathway's ferric chloride-ferricyanide reagent. In brief, ethyl acetate extract was made by extracting 20 mL of supernatant twice with an equivalent amount of solvent (ethyl acetate) at pH 2.0. Hathway's reagent was prepared by adding one mL of 0.1 M ferric chloride in 0.1 N HCl to 100 mL of distilled water, and to this, 1 mL of 0.1 M potassium ferricyanide was added [3]. For detection, an equal volume of Hathway's reagent and sample were mixed, and absorbance was measured at 560 nm for SA and 700 nm for dihydroxy phenols using sodium salicylate and 2, 3-DHBA as a standards, respectively.

S2.4.3 ACC deaminase activity

The ACC deaminase (EC 4.1.99.4) secreted by *L. adecarboylata* PAB19 was detected by spot inoculation using DF salts minimal medium [4] containing 3.0 mM of ACC as N source. Plates containing DF medium without ACC and with $(NH_4)_2SO_4$ (0.2% w/v) served as negative and positive controls, respectively. Plates were incubated for 72 h at 28±2°C. Plates containing DF medium and $(NH_4)_2SO_4$ (0.2% w/v) with or without ACC served as positive and negative and controls, respectively, and were examined daily for bacterial growth. *Mesorhizobium* LMS-1 containing pRKACC plasmid [5] was used as a positive control. ACC deaminase secreted by PAB19 in a liquid medium supplemented with different concentrations of PEG was extracted as suggested by Honma and Shimomura [6] and Penrose and Glick [7]. The quantity of α-ketobutyrate generated due to ACC deaminase activity was measured spectrophotometrically against a standard α-ketobutyrate curve. ACC deaminase activity is presented as the quantity of α-ketobutyrate released/mg of protein/h. Experiments were conducted independently three times.

S2.5 Biofilm formation and alginate production by PAB19 strain

Biofilm formation in microtiter plates in the presence or absence of PEG was assessed as described by O'Toole [8] using 1% crystal violet (CV). CV-stained biofilms were treated with 75% ethanol and absorbances were measured at 595 nm using a microtiter plate reader (Thermo Scientific Multiskan EX, UK) to determine the amount of dye retained. Controls grown without PEG were processed in a similar manner. Experiments were performed independently three times using five wells per determination.

For alginate production, cells were grown in a liquid medium containing different concentrations of PEG. An equal volume of isopropanol was added to supernatants obtained by centrifugation and filtration, and allowed to stand for 24 h at room temperature. The deacetylated alginate precipitate formed was collected by centrifugation for 10 min at 10,000 rpm, washed with graded concentrations of ethanol (70%), dried, and suspended in 1 mL of distilled water (DW). Aliquots (100 µL) of this suspension were placed in fresh tubes and the volume was made up to 1 mL with Milli-Q water. Fresh carbazole reagent (30 µL) was added to this suspension followed by 1 mL of borate sulfuric acid solution (10 mM) and mixed. The mixture was left at room temperature for 15 min and absorbance was read at 500 nm against a reagent blank. Amounts of alginate were expressed as µg/mg of wet biomass [9].

S2.6.4 Determination of photosynthetic pigment levels

Photosynthetic pigment (Chl a, Chl b, total chlorophyll, and carotenoids) levels in fresh foliage of bio-inoculated and PEG-treated *V. radiata* plants were measured as described by Arnon [10]. The pigments were extracted from fresh leaves by macerating in 80% acetone. Chlorophyll and carotenoid contents in extracts were determined by UV visible spectrophotometry (UV-2450, Shimadzu, Tokyo, Japan). Total photosynthetic pigment (Chl a, Chl b, and total chlorophyll) levels were calculated as follows:

mg chl a/g tissue = 12.7 (A_{663}) −2.69 (A_{645}) ×V/1000×W
mg chl b/g tissue = 22.9 (A_{645}) − 4.68 (A_{663}) ×V/1000×W
mg total chl/g tissue = 20.2 (A_{645}) +8.02 (A_{663}) ×V/1000×W
Carotenoid contents were determined as suggested by Krik and Allen [11] using:
Carotenoids (mg/g tissue) = (A_{480}) +0.114 (A_{663}) – 0.638 (A_{645})

Where, $A\lambda$ = absorbance at the specified wavelength λ (nm); V= final volume of chlorophyll extracted in 80% acetone, and W= fresh weight of the tissue extract.

S2.6.4 Symbiotic attributes

Symbiotic attributes in PAB19 inoculated and PEG-treated *V. radiata* plants were assayed at 50 and 80 DAS. To estimate LHb contents, nodules were crushed with a mortar and pestle in 5 mL of phosphate buffer (pH=7.4) and then filtered through two layers of cheesecloth. Turbid reddish-brown filtrates were clarified by centrifugation at 10,000 g for 30 min. Supernatants were diluted to 10 mL with phosphate buffer (pH=7.4). Each extract was divided equally into two glass tubes (5 mL/tube) and an equal amount of alkaline pyridine reagent was added to each tube. Hemachrome absorbance was read at 556 and 539 nm
after adding a few crystals of potassium hexacyanoferrate (K_3FeCN_6) or sodium dithionite ($Na_2S_2O_4$), respectively. LHb contents were calculated using the formula:
LHb content (mM) = [λ_{556}-λ_{539}] × 2D/23.4
Where D = initial dilution

S2.6.5.1.1 Proline estimation

Proline contents in the fresh foliage of *Enterobacter sp./L. adecarboxylata* PAB19 inoculated or un-inoculated *V. radiata* plants cultivated with or without PEG were determined as described by Bates et al. [12]. Briefly, 1.0 gm leaf samples were homogenized with 5 mL of 3% (w/v) aqueous sulfosalicylic acid ($C_7H_6O_6S$). The resulting homogenates were filtered through Whatman No.2 filter paper, centrifuged at 8,000 rpm for 20 min, and 2 mL aliquots were treated with acid ninhydrin (2 mL) and glacial acetic acid (2 mL) at 80°C for one hour when the reaction was terminated in an ice bath. The colored complex that formed was extracted using 4 mL toluene and absorbances were measured at 520 nm. Proline standards were prepared by dissolving proline in 3% (w/v) sulfosalicylic acid.

S2.6.5.1.2 Estimation of thiboarbituric-acid reactive substances (TBARS)

Lipid peroxidation (estimation of TBARS) in PAB19 inoculated and PEG treated leaf tissues of *V. radiata* were measured as malondialdehyde (MDA) as described earlier [13]. For the assay, 500 mg of fresh foliage was homogenized with 10 mL of tri-chloroacetic acid (TCA; 5% w/v) in an ice bath. After centrifugation (12000 ×g, 4ºC, 20 min). Equal volumes of supernatant and thiobarbituric acid (TBA; 0.67% w/v) (Hi-Media Pvt. Ltd. India) were mixed in clean, acid rinsed glass tubes and then heated at 100℃ in a water bath for 30 min when reactions were terminated by transferring the tubes to an ice-bath. After centrifugation (10000 ×g, 4℃, 10 min) the optical densities of supernatants were recorded at wavelengths (λ) of 450, 532, and 600 nm. MDA levels were calculated using the following equation using a molar extinction coefficient of 155 mM^{-1} cm^{-1}.
MDA level (μ mol/L) = 6.45× ($A_{\lambda 532}$ - $A_{\lambda 600}$) - 0.56 × $A_{\lambda 450}$
where A = Absorbance

S2.6.6 Determination of antioxidant enzyme activities

Foliage was crushed in 4 mL of enzyme extraction buffer [(50 mM phosphate buffer (pH=7.8)] containing 1mM EDTA and 2% (w/v) polyvinylpyrrolidone (PVP). For GPX (E.C. 1.11.1.7), foliage tissues (100 mg) were homogenized in tris buffer and centrifuged at 12,000 rpm for 20 min. at 4 ºC. The reaction mixture (3 mL) consisted of 100 mM phos-

phate buffer (pH=7.0), 0.1 mM EDTA and 20 mM H_2O_2. The reaction was initiated by adding 100 μL of enzyme extract. An increase in the absorbance at 470 nm due to the formation of tetra guaiacol ($\varepsilon = 26.6\ mM^{-1}\ cm^{-1}$) was recorded and GPX activity was expressed as μ mol mg $protein^{-1}\ min^{-1}$.

S2.6.6.1 Catalase (CAT)

CAT activities were measured as described by Beer and Sizer (1952) with minor modifications. The reaction mixture (3 mL) consisted of 100 mM phosphate buffer (pH=7.0), 0.1 mM EDTA, and 20 mM H_2O_2, and the reaction was initiated by adding 100 μL of the enzyme extract. Decrease in H_2O_2 absorbance were monitored at 240 nm and CAT activities were quantified using the molar extinction coefficient of H_2O_2 (36 $mol^{-1}\ cm^{-1}$).

S2.6.6.2 Superoxide dismutase (SOD)

SOD activities were assessed using the ability of SOD to inhibit the photochemical reduction in nitro-blue tetrazolium (NBT) [14]. To a reaction mixture of 3.0 mL [containing 13 mM methionine (0.1 mL), 50 mM $NaHCO_3$ (0.1 mL), 25 mM NBT (0.1 mL), 0.1 mM EDTA (0.1 mL), 50 mM buffer (2.3 mL) and 0.2 mL of enzyme extract] 2 mM riboflavin was added. The mixture was exposed to 15 W fluorescent tubes for 10 min and then incubated for 20 min in the dark. Absorbance was then measured at 560 nm.

S2.6.6.3 Glutathione reductase (GR)

Enzyme extract (0.2 mL) was added to a 2.8 mL of a reaction mixture containing buffer (30 mM GSSG, 0.8 mM NADPH, and 1% BSA). Absorbances were measured 3 min later at 340 nm [15]. Enzyme activities were expressed as μmol of ascorbate oxidized $min^{-1}\ g^{-1}$ DW.

S2.7 Rhizosphere/rhizoplane colonization by Enterobacter sp./L. adecarboxylata under water stress conditions

The root colonization efficiency of PAB19 strain *Enterobacter sp./L. adecarboxylata* was determined in the presence/absence of PEG-6000. Roots were rinsed with DDW and PBS, and colonization was initially checked by scanning electron microscopy as described by Shahid et al. [16]. Colonization of roots (expressed as CFU g^{-1} of root material) exposed to various concentrations of PEG was assessed at 50 and 80 DAS. For enumeration, rhizosphere soil tightly adhered to root surfaces was collected at 50 and 80 DAS and serially diluted into normal saline solution (NSS). Soil suspension (100 μL) from each dilution was spread on NA plates containing 30 μg/mL of rifampicin [17]. For rhizoplane colonization, 1 g of roots was rinsed with distilled water three times in a sterile Petri dish and roots were macerated and diluted in NSS. A 100 μL of the suspension was spread plated on NA plates. Plates were incubated for 48 h at 28±2ºC and CFUs/g of soil were determined by counting.

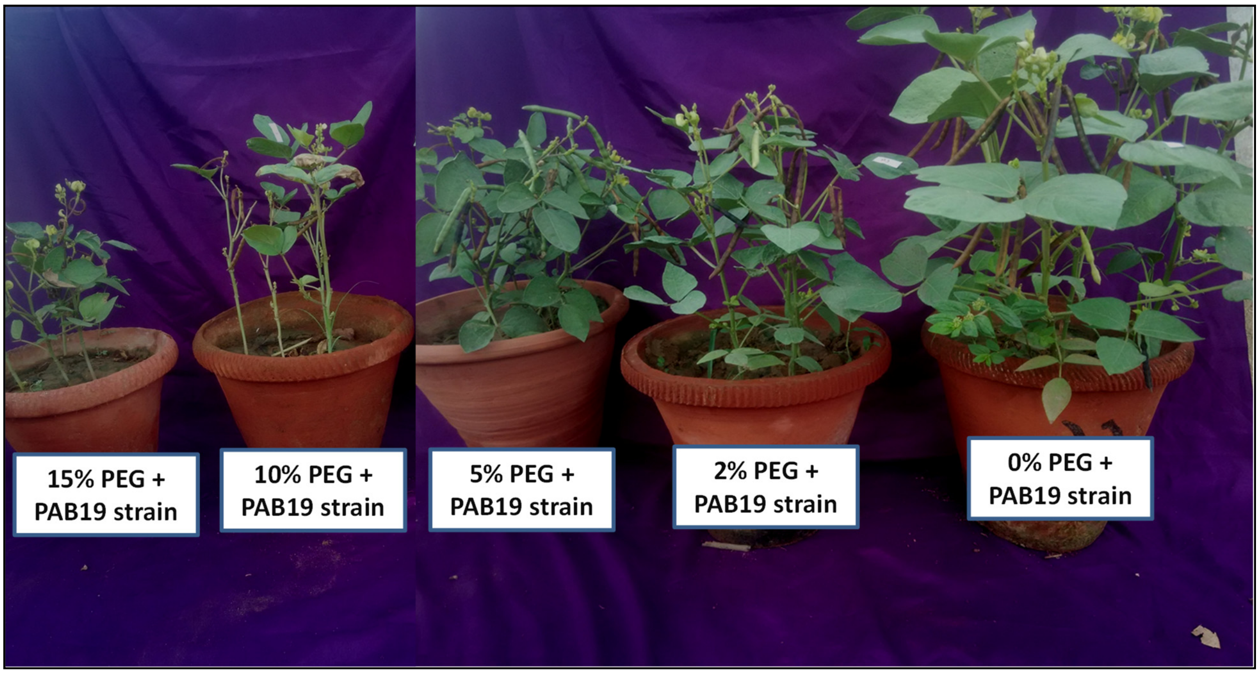

Figure S1. Representative pictures of bio-inoculated *V. radiata* plants with drought-tolerating *Enterobacter sp./L. adecarboxylata* PAB19 growing with 2%, 5%, 10%, and 15% PEG solution in the soil.

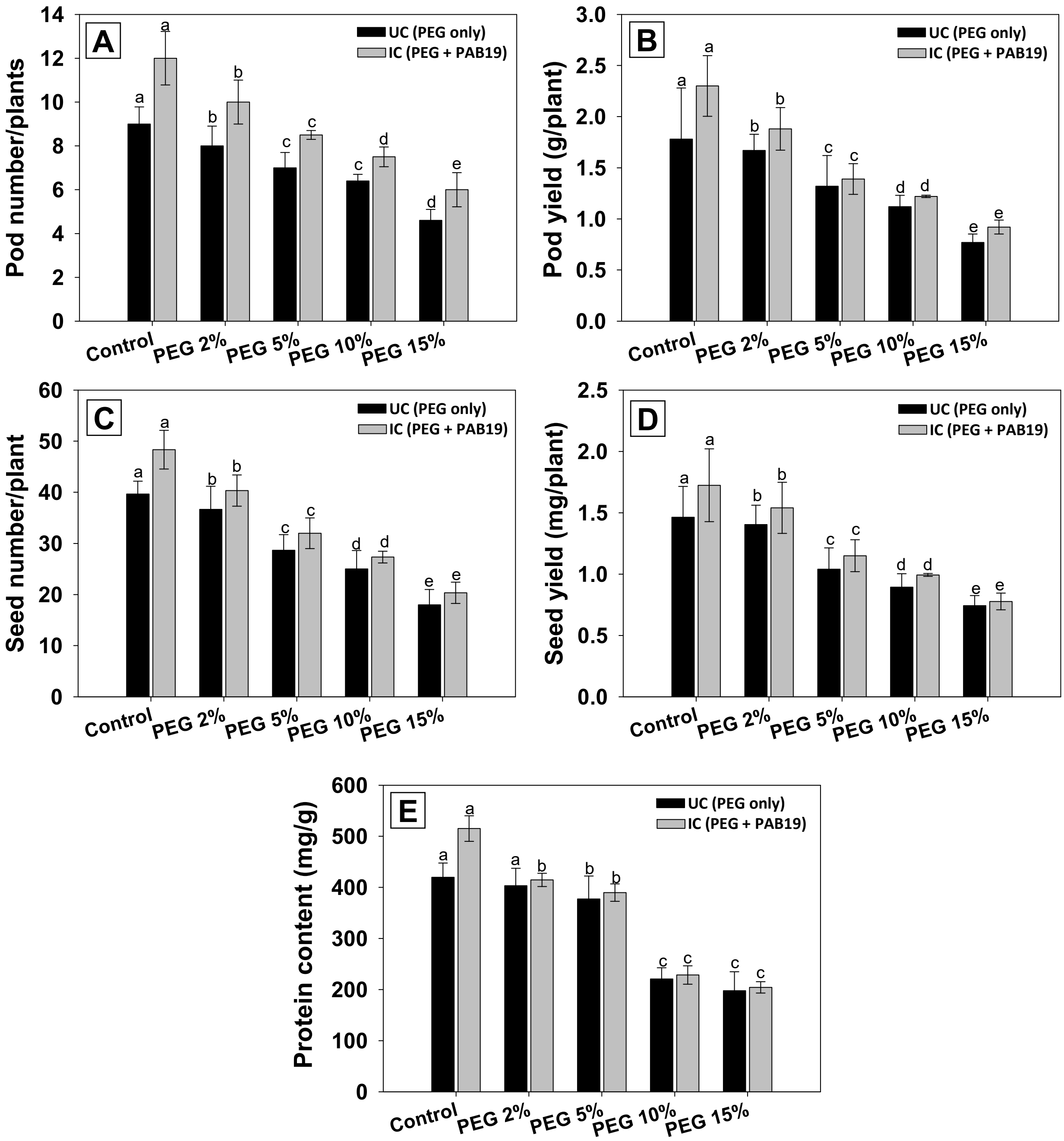

Figure S2. Impacts of PAB19 on yield parameters; pod number (A), pod yield (B), seed number (C), seed yield (D), and protein content (E) of *V. radiata* cultivated in the presence of different levels of water stress (2%, 5%, 10% and 15% PEG-6000) under green-house conditions. Bar diagrams represent the mean values of three replicates. Mean values followed by different letters are significantly different (*P* ≤0.05) as determined by the DMRT test.

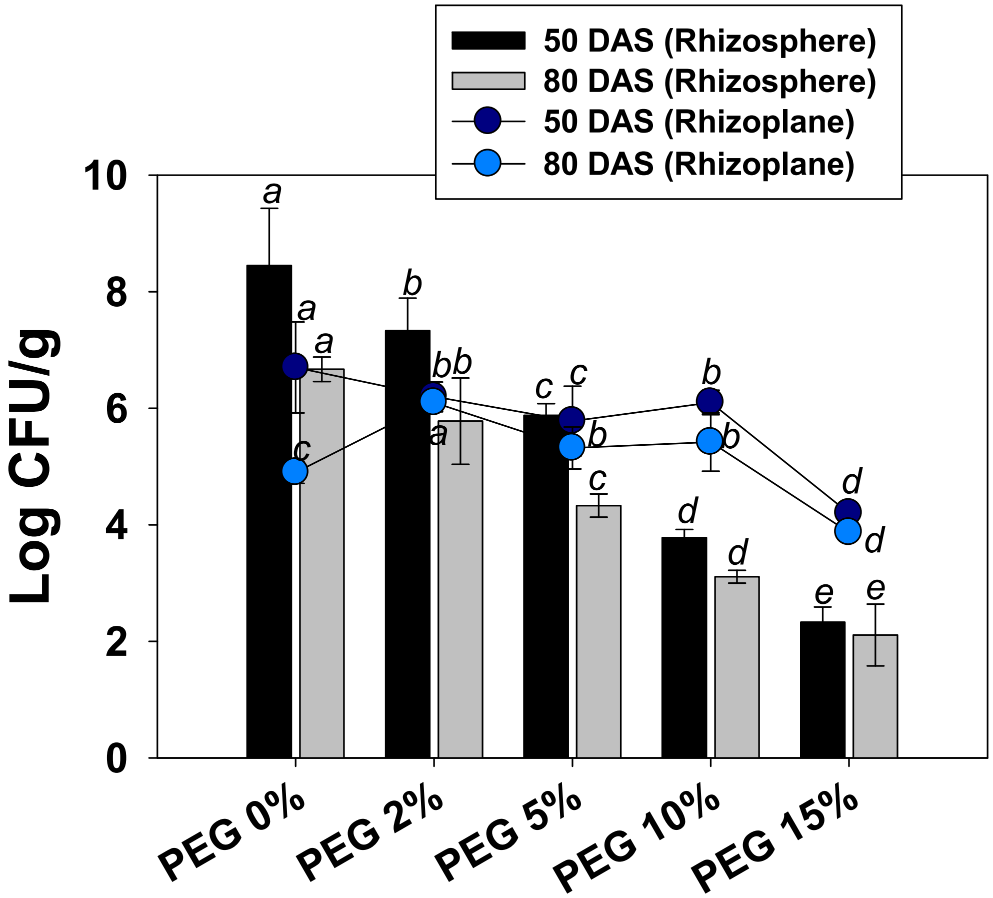

Figure S3. Colonization of the root surfaces of *V. radiata* by *Enterobacter sp./L. adecarboxylata* strain PAB19. Rhizoplane and rhizosphere colonization in the presence of different levels of PEG at two different seeding stages (50 and 80 DAS). Mean values followed by different letters are significantly different ($P \leq 0.05$) as determined by the DMRT.

References

1. Barbhaiya, H.B.; Rao, K.K. Production of pyoverdine, the fluorescent pigment of *Pseudomonas aeruginosa* PAO1. *FEMS Microbiol. Lett.* **1985**, *27*, 233–235.
2. Schwyn, B.; Neilands, J.B. Universal chemical assay for the detection and determination of siderophores. *Anal. Biochem.* **1987**, *160*, 47–56.
3. Reeves, M.W.; Pine, L.; Neilands, J.B.; Balows, A. Absence of siderophore activity in *Legionella* species grown in iron-deficient media. *J. Bacteriol.* **1983**, *154*, 324–329.
4. DWORKIN, M.; FOSTER, J.W. Experiments with some microorganisms which utilize ethane and hydrogen. *J. Bacteriol.* **1958**, doi:10.1128/JB.75.5.592-603.1958.
5. Shah, S.; Li, J.; Moffatt, B.A.; Glick, B.R. Isolation and characterization of ACC deaminase genes from two different plant growth-promoting rhizobacteria. *Can. J. Microbiol.* **1998**, doi:10.1139/w98-074.
6. Honma, M.; Shimomura, T. Metabolism of 1-aminocyclopropane-1-carboxylic acid. *Agric. Biol. Chem.* **1978**, *42*, 1825–1831.
7. Penrose, D.M.; Glick, B.R. Methods for isolating and characterizing ACC deaminase - containing plant growth - promoting rhizobacteria. *Physiol. Plant.* **2003**, *118*, 10–15.
8. O'Toole, G.A. Microtiter dish biofilm formation assay. *J. Vis. Exp. JoVE* **2011**.
9. Wozniak, D.J.; Wyckoff, T.J.O.; Starkey, M.; Keyser, R.; Azadi, P.; O'Toole, G.A.; Parsek, M.R. Alginate is not a significant component of the extracellular polysaccharide matrix of PA14 and PAO1 *Pseudomonas aeruginosa* biofilms. *Proc. Natl. Acad. Sci.* **2003**, *100*, 7907–7912.

10. Arnon, D.I. Copper Enzymes in Isolated Chloroplasts. Polyphenoloxidase in *Beta Vulgaris*. *Plant Physiol.* **1949**, *24*, 1–15, doi:10.1104/pp.24.1.1.
11. Kirk, J.T.O.; Allen, R.L. Dependence of chloroplast pigment synthesis on protein synthesis: effect of actidione. *Biochem. Biophys. Res. Commun.* **1965**, *21*, 523–530.
12. Bates, L.S.; Waldren, R.P.; Teare, I.D. Rapid determination of free proline for water-stress studies. *Plant Soil* **1973**, *39*, 205–207, doi:10.1007/BF00018060.
13. Ahmed, B.; Dwivedi, S.; Abdin, M.Z.; Azam, A.; Al-Shaeri, M.; Khan, M.S.; Saquib, Q.; Al-Khedhairy, A.A.; Musarrat, J. Mitochondrial and Chromosomal Damage Induced by Oxidative Stress in Zn^{2+} Ions, ZnO-Bulk and ZnO-NPs treated *Allium cepa* roots. *Sci. Rep.* **2017**, *7*, 40685, doi:10.1038/srep40685.
14. Dhindsa, R.S.; Plumb-Dhindsa, P.; Thorpe, T.A. Leaf senescence: correlated with increased levels of membrane permeability and lipid peroxidation, and decreased levels of superoxide dismutase and catalase. *J. Exp. Bot.* **1981**, *32*, 93–101.
15. Ahmed, B.; Rizvi, A.; Syed, A.; Elgorban, A.M.; Khan, M.S.; Al-Shwaiman, H.A.; Musarrat, J.; Lee, J. Differential responses of maize (*Zea mays*) at the physiological, biomolecular, and nutrient levels when cultivated in the presence of nano or bulk ZnO or CuO or Zn^{2+} or Cu^{2+} ions. *J. Hazard. Mater.* **2021**, *419*, 126493.
16. Shahid, M.; Ameen, F.; Maheshwari, H.S.; Ahmed, B.; AlNadhari, S.; Khan, M.S. Colonization of *Vigna radiata* by a halotolerant bacterium *Kosakonia sacchari* improves the ionic balance, stressor metabolites, antioxidant status and yield under NaCl stress. *Appl. Soil Ecol.* **2021**, *158*, doi:10.1016/j.apsoil.2020.103809.
17. Zhang, N.; Wu, K.; He, X.; Li, S.; Zhang, Z.; Shen, B.; Yang, X.; Zhang, R.; Huang, Q.; Shen, Q. A new bioorganic fertilizer can effectively control banana wilt by strong colonization with *Bacillus subtilis* N11. *Plant Soil* **2011**, *344*, 87–97.
